# Diversity, structure and sources of bacterial communities in earthworm cocoons

**DOI:** 10.1038/s41598-018-25081-9

**Published:** 2018-04-26

**Authors:** Manuel Aira, Marcos Pérez-Losada, Jorge Domínguez

**Affiliations:** 1 Departamento de Ecoloxía e Bioloxía Animal, Universidade de Vigo, Vigo, E-36310 Spain; 2 CIBIO-InBIO, Centro de Investigação em Biodiversidade e Recursos Genéticos, Universidade do Porto, Campus Agrário de Vairão, 4485-661 Vairão, Portugal; 3 Computational Biology Institute, Milken Institute School of Public Health, George Washington University, Ashburn, VA 20147 USA

## Abstract

Animals start interactions with the bacteria that will constitute their microbiomes at embryonic stage. After mating, earthworms produce cocoons externally which will be colonized with bacteria from their parents and the environment. Due to the key role bacterial symbionts play on earthworm fitness, it is important to study bacterial colonization during cocoon formation. Here we describe the cocoon microbiome of the earthworms *Eisenia andrei* and *E. fetida*, which included 275 and 176 bacterial species, respectively. They were dominated by three vertically-transmitted symbionts, *Microbacteriaceae*, *Verminephrobacter* and *Ca*. Nephrothrix, which accounted for 88% and 66% of the sequences respectively. *Verminephrobacter* and *Ca*. Nephrothrix showed a high rate of sequence variation, suggesting that they could be biparentally acquired during mating. The other bacterial species inhabiting the cocoons came from the bedding, where they accounted for a small fraction of the diversity (27% and 7% of bacterial species for *E. andrei* and *E. fetida* bedding). Hence, earthworm cocoon microbiome includes a large fraction of the vertically-transmitted symbionts and a minor fraction, but more diverse, horizontally and non-randomly acquired from the environment. These data suggest that horizontally-transmitted bacteria to cocoons may play an important role in the adaptation of earthworms to new environments or diets.

## Introduction

Animals and bacteria establish associations with different degrees of specificity, but most of their symbiotic relationships are established during the embryonic development of the animal hosts^1^. Even at this initial stage, animals must balance the advantages associated with beneficial partners while avoiding and/or controlling pathogens^1^. The environment where embryos grow is key to the future development of animals because it represents the starting point for interactions between animals and the bacteria that will constitute their microbiomes.

The study of earthworm microbiomes has mainly focused on the earthworm guts and faeces, showing that they vary greatly with diet and earthworm species^2–13^. Some studies have also suggested the existence of a nephridial microbiome comprised of at least 27 bacterial species that seems to be evolutionarily conserved^14,15^, although only 3 of them seem to be specific symbionts transferred from parents to offspring^14^. Earthworms are simultaneous hermaphrodites, and reproduction usually occurs through copulation and cross-fertilization, after which each of the mated individuals produces cocoons containing variable numbers of fertilized ova^16^. Cocoon formation in earthworms is an external process that involves production of a mucous secretion that moves forward from the clitellum to the head. Cocoons contain a nutritive albuminous fluid produced by the clitellar gland cells and gametes in which earthworm embryos develop^16^. During cocoon formation, cocoons are colonized with bacteria from two sources: the earthworm and the environment, being the contribution of the latter highly dependent on its microbial load –organic substrates, for example, have higher microbial loads than regular soils (up to 10 times higher)^2,3,6,17,18^. Moreover, previous studies have showed that cocoons of the earthworm *Eisenia fetida* harbour bacterial populations initially comprising around 10^8^–10^9^ CFU ml^−1^ ^14,19^. This initial bacterial community seems to be dominated by bacteria acquired from the earthworm, with a minor fraction coming from the environment^20,21^. It has been also shown that bacterial symbionts from gut and nephridia provide essential benefits to earthworm life (e.g., pesticide detoxification) and enhance fitness under low quality diets^22–24^. Even more important, a recent study has shown that reproduction is impaired in absence of bacterial nephridial symbionts^25^. Nonetheless, the contribution of environmental bacteria to the cocoon microbiota cannot be discarded^20,21^; hence, if we were to identify the origin of the cocoon microbiota and characterize its diversity, we need to discern the contribution of both parental and environmental sources during cocoon formation.

*Eisenia fetida* (Savigny, 1826) and *Eisenia andrei* Bouché, 1972 (Oligochaeta, Lumbricidae) are of great importance in vermicomposting, a potential source of protein for animal consumption, and as fishing bait^26^. Moreover, they are commonly used as animal models in ecotoxicology, physiology, biochemical, and genetic studies^26^. Moreover, *Eisenia andrei* and *E. fetida* are a good model to study bacterial colonization of cocoons because they are sister taxa^27,28^, inhabit organic rich substrates^29^, and their activity generates species-specific microbial profiles in the environments where they live^30^. In fact, when present, bacterial communities are richer than those of soils with no earthworms, allowing more environmental bacteria to gain access to cocoons during its formation. Here we used next-generation sequencing technology (Illumina MiSeq) and the dada2 pipeline to first describe in detail the bacterial communities (i.e. composition and diversity) living in the cocoons of the red worm *Eisenia andrei* and the tiger worm *E. fetida*, and to compare them with the bedding material in which cocoons were deposited. We then assessed whether cocoon microbiotas consist of bacterial groups of vertically transmitted symbionts plus random environmental bacteria incorporated during cocoon formation or whether there is selective recruitment during cocoon colonization.

## Results

### Composition and diversity of bacterial communities differ between cocoon and bedding materials and between earthworm species

**Figure 1:**
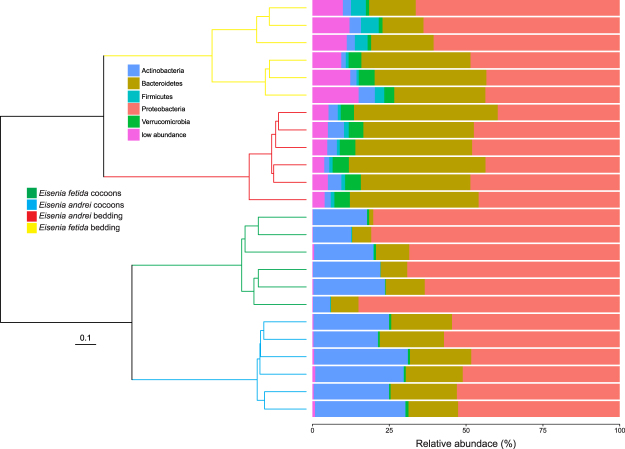
Composition of the bacterial communities in cocoons and bedding material stratified by earthworm species (*Eisenia andrei* and *Eisenia fetida*). The dendrogram represents the dissimilarity of bacterial communities at ASV level (UPGMA algorithm, unweighted UNIFRAC distances, Ward method). Bars represent the relative abundance of dominant bacterial phyla. Bacterial phyla present at low abundance (<1%) were grouped together.

Bacterial communities in cocoon and bedding material samples comprised 1,118 and 5,053 amplicon sequence variants (ASVs), respectively, from 5 main bacterial phyla (of a total of 44 bacterial phyla). Bacterial communities in cocoons of *E. andrei* and *E. fetida* and the respective bedding materials differed markedly at both phylum and ASV levels (Fig. 1). We identified two main clusters: one for bacterial communities in the cocoons and another for bacterial communities in the bedding materials. Within these two main clusters, samples were sub clustered by earthworm species (Fig. 1). Bacterial communities in all samples (cocoons and bedding materials) were mainly comprised of ASVs belonging to the phyla *Proteobacteria*, *Bacteroidetes* and *Actinobacteria*, which contributed to 93% of the sequences, which decreased to 89% when removing the contribution of the three vertical transmitted nephridial symbionts (Table 1). *Proteobacteria* relative abundance was significantly higher in cocoon samples than in bedding samples, but only cocoons of *E. fetida* had more *Proteobacteria* than bedding samples (57%, Supplementary Figure 2). *Bacteroidetes* were significantly more abundant in bedding samples than in cocoon samples, but only bedding of *E. fetida* had more *Bacteroidetes* than cocoons (135%, Supplementary Figure 2). *Actinobacteria* were more abundant in *E. andrei* than in *E. fetida* samples, although there were only higher in bedding than cocoon samples in *E. fetida* (470%, Supplementary Figure 2). *Firmicutes* and *Planctomycetes* showed no differences between cocoon and bedding samples for *E. andrei*, and higher abundance in bedding than in cocoon for *E. fetida* (395% and 243% respectively, Supplementary Figure 2). *Acidobacteria* were always more abundant in bedding than in cocoon samples, although differences were only significant for *E. fetida* (951%, Supplementary Figure 2). Some bacterial phyla were more abundant in *E. andrei* samples (*Verrucomicrobia* and *Planctomycetes*) while others were more abundant in *E. fetida* samples (*Firmicutes* and *Acidobacteria*; Supplementary Figure 2). Bacterial communities in the bedding materials of earthworms *E. andrei* and *E. fetida* are mainly comprised of different ASVs from the phyla *Proteobacteria*, *Bacteroidetes* and *Actinobacteria* (19 out of 20), suggesting an earthworm species-specific composition (Supplementary Table 1). Remarkably, we did not find any ASV common to cocoon and bedding material samples within the 20 most abundant ASVs (Supplementary Table 1).

**Table 1:** Relative abundance (%, mean ± SE) and taxonomy (phylum and genus or most accurate taxonomy) of three vertical transmitted nephridial symbionts found in bacterial communities of cocoons and bedding from the earthworm species *Eisenia andrei* and *Eisenia fetida*.

Amplicon sequence variant (ASV)	*Eisenia andrei*		*Eisenia fetida*	
	cocoon	bedding	cocoon	bedding
ASV1, Proteobacteria, *Verminephrobacter*	0	0	44.68 ± 4.77	0
ASV2, Proteobacteria, *Verminephrobacter*	45.85 ± 0.89	0.03 ± 0.02	0 ± 0	0
ASV3, Actinobacteria, Microbacteriaceae	25.95 ± 1.49	0	16.57 ± 2.72	0
ASV4, Bacteroidetes, *Candidatus* Nephrothrix	16.51 ± 1.25	0	1.70 ± 1.38	0
ASV11, Bacteroidetes, *Candidatus* Nephrothrix	0	0	2.13 ± 0.88	0
ASV12, Bacteroidetes, *Candidatus* Nephrothrix	0.05 ± 0.03	0	1.65 ± 0.75	0
ASV41, Bacteroidetes, *Candidatus* Nephrothrix	0	0	0.47 ± 0.44	0
ASV177, Proteobacteria, *Verminephrobacter*	0.14 ± 0.07	0	0	0
ASV561, Proteobacteria, *Verminephrobacter*	0	0	0.05 ± 0.02	0
ASV758, Proteobacteria, *Verminephrobacter*	0.03 ± 0.02	0	0	0
ASV1013, Proteobacteria, *Verminephrobacter*	0	0	0.02 ± 0.02	0
ASV3848, Proteobacteria, *Verminephrobacter*	0	0	0.01 ± 0.01	0
ASV4096, Proteobacteria, *Verminephrobacter*	0	0	<0.0001	0
ASV4172, Proteobacteria, *Verminephrobacter*	0	0	<0.0001	0

**Figure 2:**
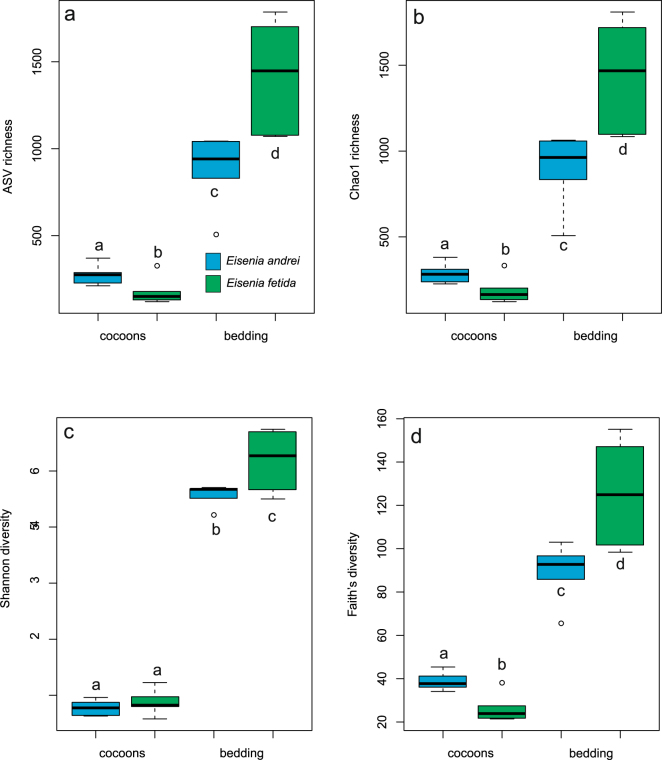
Box plots of alpha-diversity estimates of bacterial communities in cocoons and bedding material of the earthworms *Eisenia andrei* and *Eisenia fetida*. (**a**) Observed OTU richness, (**b**) estimated taxonomic richness (Chao 1), (**c**) taxonomic diversity (Shannon index) and (**d**) phylogenetic diversity (Faith’s PD). Different letters over box plots indicate significant differences between treatments (Tukey HSD test, FDR corrected).

**Figure 3:**
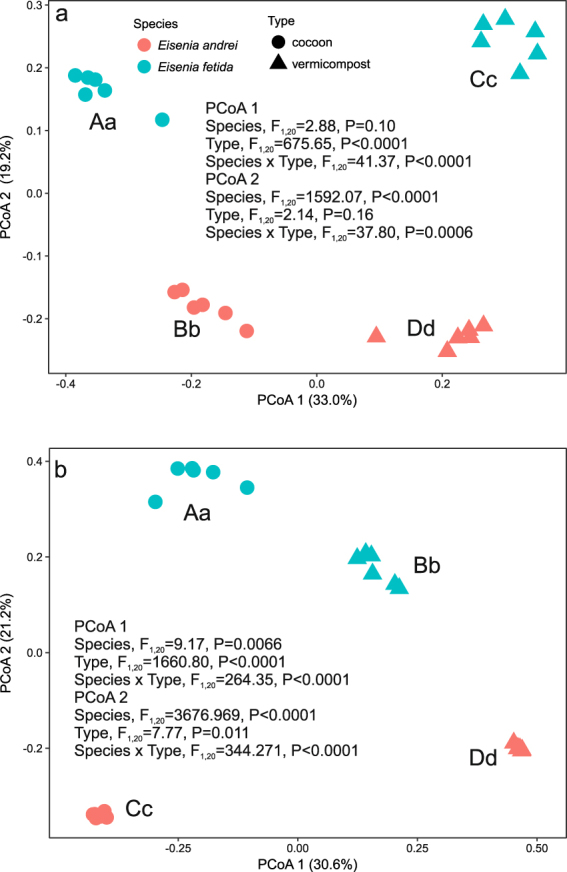
Principal coordinate analyses (PCoAs) of (**a**)) unweighted UniFrac and (**b**) Jaccard β-diversity of bacterial communities in cocoons and bedding material of the earthworms *Eisenia andrei* and *E. fetida*. Different capital and lower case letters near the symbols indicate significant differences between treatments in PCoA 1 and 2 scores respectively (Tukey HSD test, FDR corrected).

Bacterial communities in cocoons were less diverse (taxonomic and phylogenetic α-diversity) than bacterial communities in the bedding material in which they were deposited (Fig. 2). Interestingly, this difference was always two times higher for *E. fetida* than for *E. andrei* samples, resulting in significant interactions between earthworm species and type of sample for all variables except for the Shannon index (Fig. 2; *P* < 0.001). The taxonomic and phylogenetic composition of bacterial communities in the cocoons differed significantly (Fig. 3; Supplementary Figure 4) from that of the bacterial communities in the bedding materials, and also between earthworm species. Cocoon and bedding material samples differed clearly in the PCoA 1 of all distance measures (Fig. 3, Supplementary Figure 3, P < 0.0001 for the four distance matrices; randomization test, *P* = 0 for the four distance matrices; Supplementary Figure 4). This finding remained consistent even when considering only the most abundant ASVs (914 from a total of 5,411 ASVs; Supplementary Figure 5, randomization test, P = 0 for the four distance matrices; Supplementary Figure 6). Furthermore, differences in dissimilarity of samples across PCoA 1 between the two earthworm species yielded a significant interaction between species and type of sample (Fig. 3, Supplementary Figure 3; P < 0.0001 except for weighted unifrac). Interestingly, we found that samples from the two earthworm species were very different in relation to PCoA 2 (Fig. 3, Supplementary Figure 3, P < 0.0001 for the four distance matrices), and that a contrasting pattern of separation between cocoon and bedding material samples between species yielded a significant interaction between species and type of sample (Fig. 3, Supplementary Figure 3, P < 0.001 except for Jaccard). Moreover, analysis of the bacterial communities in subsets of samples for each of the most abundant phyla revealed that the pattern of differentiation between cocoon and bedding material samples (more or less markedly) in PCoA 1 was also consistent across most of the phyla and all distances used (Supplementary Figures 7–10). The same pattern appeared in some cases in PCoA 2 (such as with *Planctomycetes*; Supplementary Figures 7–10).

### Bacterial nephridial symbionts vary between earthworm cocoons

The bacterial communities in cocoons were dominated by ASVs corresponding to known nephridial bacterial symbionts, which accounted for 88% of the sequences in *E. andrei* (ASV2-ASV4 and ASV177) and 66% of the sequences in *E. fetida* (ASV1, ASV3, ASV4, ASV11, ASV12 and ASV41) (Supplementary Table 1). The most abundant ASVs (ASV2 and ASV1 for *E. andrei* and *E. fetida*, respectively) were classified as *Verminephrobacter*, a known symbiont of earthworm nephridia. ASV1 was also found in the bedding samples (Table 1). Interestingly, each earthworm species showed a specific *Verminephrobacter* ASV, although with similar abundance. Another six ASVs (ASV561, ASV758, ASV1013, ASV3848, ASV4096 and ASV4172) were also classified as *Verminephrobacter* (Table 1) and appeared in very low numbers in cocoons and never in bedding samples (Table 1). Two of them appeared only in cocoons of *E. andrei* (ASV177 and ASV758), whereas the other four were exclusive of cocoons of *E. fetida* (ASV1013, ASV3848, ASV4096 and ASV4172) (Table 1). Another important contributor to the cocoon microbiota was ASV3, which we classified as *Microbacteriaceae*-symbiont (see Methods), another known nephridial bacterial symbiont. For confirmation, we conducted a BLAST search with sequences for this and other 26 ASVs classified as Microbacteriaceae-unclassified and none of them were classified as the Microbacteriaceae symbiont described by Davidson *et al*.^14^. Opposed to *Verminephrobacter* ASVs, this symbiont, ASV3, was the same for the two earthworm species, and appeared in higher abundance in *E. andrei* cocoons, and not in bedding samples (Table 1). Other important ASVs found in cocoon samples were those classified as “*Candidatus* Nephrothrix”, which is another known symbiont associated with earthworm nephridia^30^. We found two ASVs (ASV4 and ASV12) that appeared in *E. andrei* and *E. fetida* cocoons (Table 1). Interestingly, the most abundant ASVs for *E. andrei* and for *E. fetida* were ASV4 and ASV11, respectively. ASV11 and ASV41 were exclusive *E. fetida*. There was not any ASV exclusive for *E. andrei*, and the four ASVs were not present in the bedding (Table 1).

Since bacterial communities from earthworm nephridia could comprise up to 27 bacterial species^14,15^, we would expect that any other bacteria present in earthworm nephridia would be also present in cocoons. Opposed to the three vertical transmitted symbionts, none of these bacteria seems to be vertically transmitted, and their presence could be associated with horizontal transmission. Therefore, we searched the bacterial communities from cocoons for ASVs representing bacteria associated with earthworm nephridia^14,15^. We found 202 ASVs whose taxonomy matched those of described nephridial bacteria^14,15^, but only 95 were possible symbionts because there were present in cocoon samples (Supplementary Table 2). Most of ASVs found in cocoons were also present in bedding samples at higher (i.e. ASV228, *Achromobacter*) or lower abundances (i.e. ASV356, *Devosia*). Although these ASVs showed low relative abundances (<1% each), they comprised roughly 2 and 11% of the sequences in *E. andrei* and *E. fetida* cocoons, and 10 and 6% of the bedding samples (Supplementary Table 2). Some ASVs were shared by the two earthworm species, like ASV10 (*Chitinophaga*), ASV18 (*Devosia*) or ASV25 (*Achromobacter*), whereas other were earthworm species-specific like ASV1246 (*Azospira*), ASV942 (*Paenibacillus*) or ASV301 (*Pedobacter*, Supplementary Table 2). In fact, for each bacterial genus, there were earthworm specific ASVs except for *Achromobacter*, *Bordetella*, *Bradyrhizobium* and *Phyllobacterium* (Supplementary Table 2).

## Discussion

We provide a detailed description of the bacterial communities in cocoons of two detritivorous earthworm species, the redworm *Eisenia andrei* and the tiger worm *Eisenia fetida*. The communities were mainly formed by ASVs belonging to the bacterial phyla *Proteobacteria*, *Bacteroidetes* and *Actinobacteria*. The cocoons and bedding materials of *E. andrei* showed the same abundances for these bacterial phyla, whereas the cocoons of *E. fetida* were richer in *Proteobacteria* and lower in *Actinobacteria* and *Bacteroidetes* than the bedding materials. Proliferation or selective recruitment of *Actinobacteria* may be associated with inhibitory activity observed in earthworm cocoons^14,31–33^. The inhibitory activity may be involved in the protection of embryos, as in some insects and viviparous sponges (reviewed in 14). However, the two earthworm species differed in the amount of *Actinobacteria* colonizing their cocoons, which may be related to the higher hatchability of cocoons of *E. andrei* compared to *E. fetida*^25^. The composition of the bacterial communities in *E. andrei* and *E. fetida* cocoons resembles that of the gut microbiome of *E. andrei*, which is governed by OTUs (not ASVs) from the phyla *Proteobacteria*, *Actinobacteria*, *Bacteroidetes* and *Firmicutes*^2,3^.

The bacterial communities in cocoons were dominated by four bacterial ASVs corresponding to three known symbionts associated with earthworm nephridia, i.e. *Verminephrobacter*, “*Ca*. Nephrothrix” and *Microbacteriaceae*^14,34–36^. These four ASVs comprised 88% and 66% of the bacterial sequences in cocoons of *E. andrei* and *E. fetida*, respectively, as previously revealed by T-RFLPs and cloning and sequencing for *E. fetida* cocoons^14^. However, our findings regarding other ASVs present in earthworm cocoons are not consistent with previous reports. For example, Davidson *et al*.^14^ reported the presence of *Herbaspirillum* and some close relatives of *Sphingomonas*, *Stenotrophomonas*, *Enterobacter Janthinobacterium, Bordetella, Klebsiella*, *Terriglobus*, *Chitinophaga, Flavisolibacter* and *Pseudomonas*. Of these, we only detected *Chitinophaga* and *Pseudomonas* within the twenty most abundant ASVs. We also found ASVs classified as *Sphingomonas* and *Stenotrophomonas*, but niether *Flavisolibacter* and *Klebsiella* (both present in the bedding material), nor *Janthinobacterium*, *Terriglobus* or *Herbaspirillum*. Different outcomes between this and Davidson *et al*.’*s* study^14^ are likely due to the higher resolution of the NGS technique used here compared to T-RFLPs and the bedding material used (grape marc versus mixed coir with oat meal and coffee grounds), which should provide some of the components of the bacterial communities in cocoons. We also found that cocoons contained representatives of other known bacteria present in nephridia of several species of earthworms^15^, such as *Devosia*, *Pedobacter*, *Dyadobacter*, *Paenibacillus*, *Rhizobium*, *Mesorhizobium*, *Achromobacter*, *Bosea*, *Azospira*, *Azospirillum*, *Bradyrhizobium* and *Phyllobacterium*. This suggests that the composition of bacterial communities associated with nephridia may be evolutionarily conserved, as shown for vertically transmitted bacterial symbionts^34,37^. This suggests a strong pattern of selection due to the specific functions that these bacteria can provide to earthworms, like pesticide detoxification and improved fitness in response to low quality diets^22–24^ or reproduction^25^.

The earthworm cocoons contained a diverse bacterial community, particularly the *Eisenia andrei* cocoons (275 ASVs compared to 177 ASVs in *E. fetida* cocoons). A previous description of bacterial communities of cocoons did not include any measurement of bacterial diversity^9^, thus we do not know whether our diversity estimates are high or low. We should, however, expect a substantial increase in diversity estimated via NGS relative to T-RFLP, as previously reported in a comparison of both techniques in soil samples^38,39^.

The absence in the bedding materials of the four most abundant nephridial symbiont ASVs in the cocoons (only ASV2 was present at very low abundance) rejects the possibility of cocoon colonization by bacteria from the environment during cocoon formation, as suggested for “*Ca*. Nephrothrix” in earthworm dense populations^34^. Interestingly, the four and nine “*Ca*. Nephrothrix” and *Verminephrobacter* ASVs indicate that these two bacterial species could have been acquired vertically from the two parents during mating. This has been suggested for *Verminephrobacter* in the earthworm *Aporrectodea tuberculata*, which may also have increased symbiont diversity^40^. Besides biparental transmission, our sampling protocol (composite samples of five cocoons) may have increased the number of ASVs; we did not find, however, a similar high increase in ASVs for the other three symbiotic species, although, this could be also due to lack of resolution of the 16 S fragment sequenced. It is also possible that bacteria from nephridia, skin or coelom may be present in the environment simply as a result of earthworm activities (e.g. excretion, mating and release of mucus to maintain skin moisture), thus increasing their presence with earthworm density. The fact of that most ASVs shared by cocoons and bedding materials were more abundant in the latter (as opposed to *Verminephrobacter*, “*Ca*. Nephrothrix” and *Microbacteriaceae* ASVs) seem to support this idea.

Together all these findings indicate that some members of the bacterial communities in cocoons come from the environment where the cocoons were laid^21^. It is not clear whether these ASVs were selected before or after entering the cocoon, but under no microbial selection, one would expect a higher frequency in the cocoons of the most abundant ASVs found in the bedding material. Selection within the cocoon may result from strong bacteriostatic activity against Gram positive and negative bacteria found in cocoon albumen^41^. This is supported by the analysis of β-diversity, which showed that bacterial communities in cocoons are not random assemblages derived from bedding materials. This same pattern was observed when the analysis was restricted to the most abundant ASVs in the data set, which suggests that bacterial selection does not depend on the less abundant ASVs or on different patterns of presence/absence of ASVs between cocoons and bedding materials. Moreover, this selection seems to be species-specific, as revealed by our PCoA plots. It is not clear whether the environmental contribution of bacterial communities could be more similar for the cocoons of the two earthworm species than for the respective vermicomposts, as we found that the bacterial communities from the two vermicomposts were different.

We provide a detailed description of the structure of bacterial communities in earthworm cocoons at their initial stage. Although we found that most bacteria in the cocoons were also present in the bedding material (99% of ASVs for both earthworm species), the experimental design did not allow us to specify whether these ASVs were derived from the environment or the earthworms. Comparison of the bacterial communities in cocoons of the same species in different environments will enable us to determine the contribution of environmental bacteria to the bacterial communities in the cocoons. Further studies are needed to establish the specific composition of different earthworm microbiomes (nephridia, skin-mucus and coelom) as well as to ascertain which bacteria are derived from each microbiome. Longitudinal studies would also help to clarify the dynamics of embryo colonization.

## Methods

### Experimental design

To study the diversity of the bacterial communities in earthworm cocoons we selected the earthworms *Eisenia andrei* and *E. fetida*. They are sister species^27,28^ commonly found in organic rich substrates, frequently in co-occurence^29^, and their feeding activities generate species-specific microbial profiles in the environments where they live^30^. Such differences in microbial diversity should provide different sources of colonizing bacteria for cocoons.

We used *Eisenia andrei* and *Eisenia fetida* cultures established in our laboratory in 2012. Cultures were fed periodically and exclusively with the same grape marc and following the same feeding scheme. Grape marc is similar to the natural feeding substrates (decomposing vegetal material) these two species live on. The bedding substrate was vermicompost from grape marc, and since each earthworm species produces a species-specific microbial profile of their environments^30^, the two beddings should be microbiologically different. Bedding material is partially removed once a year when there is not space in the earthworm culture.

### Sampling and DNA extraction

We collected fresh earthworm cocoons (no more than 1 day old) from the 5-year-old cultures and inspected them under a dissection microscope to confirm the absence of developing earthworms^14,21^. Cocoons containing developing earthworms were discarded. We also obtained six independent composite samples of the bedding material where the cocoons were deposited. We did not include grape marc in this analysis because it was the same for all earthworm cultures and because cocoons were deposited in the layer of bedding material from the cultures.

Cocoons of each earthworm species were processed as six independent composite samples of five cocoons. The cocoons were first washed in tap water and then in sterile distilled water; they were then surface sterilized by sequential immersion in 95% (vol/vol) ethanol (5 s), 0.5% NaOCl (2 min), and 70% vol/vol ethanol (2 min), and finally rinsed three times (1 min each time) in sterile distilled water^42^. The washed cocoons were placed in Eppendorf tubes and homogenized with the aid of a micropestle under sterile conditions in a laminar flow hood. DNA was then extracted using a DNeasy Blood and Tissue kit (QIAGEN, Valencia, California). Bedding substrate was extracted using the PowerSoil DNA Isolation kit (MoBio Laboratories Inc., Carlsbad, California). Six replicates of each sample type (i.e. cocoons and bedding material) were processed for each earthworm species. DNA extractions were performed according to the manufacturer’s protocol. All laboratory procedures were performed under a laminar flow hood to prevent contamination of the samples with microorganisms from the surrounding environment.

### Amplification, sequencing and analysis of 16S rRNA genes

We amplified and sequenced a fragment of the 16 S rRNA gene covering the V4 region by using a dual-index sequencing strategy^43^ and an Illumina MiSeq genome sequencer (Genomics Core Facility, Universitat Pompeu Fabra).

The pipeline DADA2 (version 1.6) was used to infer the amplicon sequence variants (ASVs) present in each sample^44^. Exact sequence variants provide a more accurate and reproducible description of amplicon-sequenced communities than operational taxonomic units (OTUs) defined at a constant level (97% or other) of sequence similarity^45^. Bioinformatics processing largely followed the DADA2 tutorial (https://benjjneb.github.io/dada2/tutorial.html). Forward and reverse read pairs were trimmed and filtered, with forward reads truncated at 220 nt and reverse reads at 120 nt, no ambiguous bases allowed, and each read required to have less than two expected errors based on their quality scores. ASVs were independently inferred from the forward and reverse of each sample using the run-specific error rates, and then read pairs were merged. Chimeras were identified for each sample and removed if identified in a sufficient fraction of the samples. Taxonomic assignment was performed against the Silva v128 database using the implementation of the RDP naive Bayesian classifier available in the dada2 R package (min boot 80)^46,47^. We added to the Silva database sequences from *Candidatus* Nephrothrix and *Microbacteriaceae* bacterial sequences to look for these nephridial bacterial symbionts. We remove singleton and doubleton ASVs. A total of 2,840,568 sequences (mean: 118,357, SD: 37,941) passed all quality filters and were assigned to ASVs (7429 and 5411 before and after rarefaction, respectively, without singletons). We subsampled all samples to 28,677 sequences per sample to normalize the number of sequences. Rarefaction curves indicated that the sampling depth was optimal for cocoon and bedding material samples (Supplementary Figure 1). Sequence data have been uploaded to the GenBank SRA database under accession SRP095415.

### Statistical analysis

All samples were subsampled to the smallest sample size (28,677 sequences) to remove the effect of sample size bias on community composition. An approximately maximum-likelihood phylogenetic tree was inferred using FastTree 2.1^48^. Taxonomic alpha-diversity was calculated as the number of observed ASVs (Sobs), and by the Shannon diversity and Chao1 richness indexes. Phylogenetic diversity was calculated as Faith’s phylogenetic diversity^49^. The effect of earthworm species (*E. andrei* and *E. fetida*) and type of sample (cocoon or bedding material) on both taxonomic and phylogenetic alpha-diversity of bacterial communities was analyzed using linear models with earthworm species and type of sample as fixed factors. The normality of residuals and homogeneity of variance across groups was checked for each variable. Tukey’s test was used for post-hoc comparisons, and Benjamini–Hochberg FDR was used as a multiple test correction method by using the multcomp library^50^. We used the same model to test for differences in the relative abundances of bacterial phyla. We used t-tests with Benjamini–Hochberg FDR correction for the analysis of the relative abundance of dominant ASVs in the bacterial communities of cocoons and bedding materials in each earthworm species.

Taxonomic beta-diversity at the ASV level was estimated as the difference in the composition of the bacterial taxonomic community between cocoon and bedding material samples. This was done by coupling principal coordinate analysis (PCoA) with distance matrixes that take the abundance of ASVs into account (Bray–Curtis) or not (Jaccard). Phylogenetic beta-diversity was also estimated by PCoA of weighted (considering abundance of ASVs) and unweighted unifrac matrix distances^51^ by using the phyloseq library^52^. We analyzed differences in beta-diversity by using linear models, with earthworm species and type of sample as fixed factors over PCoA scores of first two axes. Tukey’s test was used for post-hoc comparisons and Benjamini–Hochberg FDR was used as multiple test correction method. We used Monte Carlo randomization analysis to determine whether or not bacterial communities in cocoons are random assemblages of bacterial communities present in bedding material. We randomly allocated observations within type of sample (cocoon or bedding material) without replacement (n = 10,000 simulations). Randomization tests are robust for analysis of small samples^53^. All analyses were performed with R 3.1 (2014)

## Electronic supplementary material

Supplementary Material
