# Supplementary material for: Diversity, structure and sources of bacterial communities in earthworm cocoons
Source: Sci Rep. 2018 Apr 26;8:6632. doi: 10.1038/s41598-018-25081-9 (PMC5919978; doi:10.1038/s41598-018-25081-9)
Supplement: Supplementary file 1 — Supplementary Material [file 41598_2018_25081_MOESM1_ESM.pdf]

Diversity, structure and sources of bacterial communities in earthworm cocoons

Manuel Aira, Marcos Pérez-Losada and Jorge Domínguez

Supplementary Table 1. Relative abundance (%, mean±SE) and taxonomy of 20 most abundant ASVs of bacterial communities of cocoons and bedding of earthworm species Eisenia andrei and *Eisenia fetida.* ASVs with matching taxonomy of known vertical transmited nephridial bacterial symbionts are marked in bold.

ASV	Type of sample	Species	Phylum	Class	Order	Family	Genus	mean	S.E.
**ASV2**	**cocoon**	***Eisenia andrei***	**Proteobacteria**	**Betaproteobacteria**	**Burkholderiales**	**Comamonadaceae**	***Verminephrobacter***	**45.85**	**0.89**
**ASV3**	**cocoon**	***Eisenia andrei***	**Actinobacteria**	**Actinobacteria**	**Micrococcales**	**Microbacteriaceae**	**unclassified**	**25.95**	**1.49**
**ASV4**	**cocoon**	***Eisenia andrei***	**Bacteroidetes**	**Cytophagia**	**Cytophagales**	**Cytophagaceae**	***Candidatus_Nephrothrix***	**16.51**	**1.25**
ASV25	cocoon	*Eisenia andrei*	Proteobacteria	Betaproteobacteria	Burkholderiales	Alcaligenaceae	*Achromobacter*	0.89	0.89
ASV7	cocoon	*Eisenia andrei*	Proteobacteria	Alphaproteobacteria	Rhizobiales	Rhizobiaceae	*Neorhizobium*	0.47	0.35
ASV44	cocoon	*Eisenia andrei*	Proteobacteria	Gammaproteobacteria	Enterobacteriales	Enterobacteriaceae	unclassified	0.37	0.24
ASV35	cocoon	*Eisenia andrei*	Proteobacteria	Betaproteobacteria	Burkholderiales	Comamonadaceae	*Xenophilus*	0.32	0.19
ASV30	cocoon	*Eisenia andrei*	Bacteroidetes	Cytophagia	Cytophagales	Cytophagaceae	*Chryseolinea*	0.27	0.05
ASV71	cocoon	*Eisenia andrei*	Proteobacteria	Deltaproteobacteria	Oligoflexales	Oligoflexaceae	*Oligoflexus*	0.25	0.14
ASV91	cocoon	*Eisenia andrei*	Bacteroidetes	Cytophagia	Cytophagales	Cytophagaceae	unclassified	0.24	0.14
ASV73	cocoon	*Eisenia andrei*	Proteobacteria	Deltaproteobacteria	Myxococcales	Blfdi19	unclassified	0.22	0.05
ASV92	cocoon	*Eisenia andrei*	Bacteroidetes	Flavobacteriia	Flavobacteriales	Flavobacteriaceae	*Chryseobacterium*	0.19	0.14
ASV60	cocoon	*Eisenia andrei*	Proteobacteria	Alphaproteobacteria	Rhizobiales	Rhizobiaceae	*Shinella*	0.19	0.02
ASV155	cocoon	*Eisenia andrei*	Verrucomicrobia	Opitutae	Opitutae_vadinHA64	unclassified	unclassified	0.16	0.03
ASV133	cocoon	*Eisenia andrei*	Proteobacteria	Alphaproteobacteria	Rhizobiales	Hyphomicrobiaceae	*Devosia*	0.16	0.04
**ASV177**	**cocoon**	***Eisenia andrei***	**Proteobacteria**	**Betaproteobacteria**	**Burkholderiales**	**Comamonadaceae**	***Verminephrobacter***	**0.14**	**0.07**
ASV157	cocoon	*Eisenia andrei*	Bacteroidetes	Sphingobacteriia	Sphingobacteriales	Chitinophagaceae	unclassified	0.14	0.06
ASV118	cocoon	*Eisenia andrei*	Bacteroidetes	Cytophagia	Cytophagales	Cytophagaceae	*Leadbetterella*	0.13	0.05
ASV78	cocoon	*Eisenia andrei*	Proteobacteria	Alphaproteobacteria	Rhizobiales	unclassified	unclassified	0.12	0.11
ASV115	cocoon	*Eisenia andrei*	Proteobacteria	Alphaproteobacteria	Rhodobacterales	Rhodobacteraceae	unclassified	0.12	0.05
ASV10	bedding	*Eisenia andrei*	Bacteroidetes	Sphingobacteriia	Sphingobacteriales	Chitinophagaceae	*Chitinophaga*	4.53	0.25
ASV13	bedding	*Eisenia andrei*	Bacteroidetes	Sphingobacteriia	Sphingobacteriales	Saprospiraceae	unclassified	3.54	0.23
ASV14	bedding	*Eisenia andrei*	Proteobacteria	Gammaproteobacteria	Pseudomonadales	Moraxellaceae	unclassified	3.23	0.18
ASV16	bedding	*Eisenia andrei*	Proteobacteria	Gammaproteobacteria	Alteromonadales	Alteromonadaceae	*Alishewanella*	2.96	0.33
ASV22	bedding	*Eisenia andrei*	Bacteroidetes	Cytophagia	Cytophagales	Cyclobacteriaceae	*Algoriphagus*	2.92	0.19
ASV19	bedding	*Eisenia andrei*	Proteobacteria	Gammaproteobacteria	Xanthomonadales	Xanthomonadaceae	unclassified	2.67	0.21
ASV27	bedding	*Eisenia andrei*	Proteobacteria	Gammaproteobacteria	Pseudomonadales	Moraxellaceae	unclassified	1.57	0.14
ASV28	bedding	*Eisenia andrei*	Proteobacteria	Alphaproteobacteria	Sphingomonadales	Sphingomonadaceae	*Novosphingobium*	1.45	0.12
ASV26	bedding	*Eisenia andrei*	Bacteroidetes	Cytophagia	Cytophagales	Cytophagaceae	*Ohtaekwangia*	1.41	0.11
ASV32	bedding	*Eisenia andrei*	Bacteroidetes	Bacteroidia	Bacteroidales	Bacteroidaceae	*Bacteroides*	1.33	0.24
ASV31	bedding	*Eisenia andrei*	Verrucomicrobia	Verrucomicrobiae	Verrucomicrobiales	Verrucomicrobiaceae	*Haloferula*	1.23	0.06
ASV36	bedding	*Eisenia andrei*	Bacteroidetes	Sphingobacteriia	Sphingobacteriales	Chitinophagaceae	*Flavitalea*	1.09	0.16
ASV33	bedding	*Eisenia andrei*	Bacteroidetes	Sphingobacteriia	Sphingobacteriales	Chitinophagaceae	*Filimonas*	1.01	0.09
ASV39	bedding	*Eisenia andrei*	Proteobacteria	Gammaproteobacteria	unclassified	unclassified	unclassified	0.92	0.03
ASV47	bedding	*Eisenia andrei*	Bacteroidetes	Sphingobacteriia	Sphingobacteriales	Sphingobacteriaceae	*Olivibacter*	0.90	0.12
ASV29	bedding	*Eisenia andrei*	Proteobacteria	Gammaproteobacteria	Pseudomonadales	Pseudomonadaceae	*Pseudomonas*	0.89	0.19
ASV51	bedding	*Eisenia andrei*	Proteobacteria	Betaproteobacteria	Rhodocyclales	Rhodocyclaceae	*Uliginosibacterium*	0.86	0.12
ASV48	bedding	*Eisenia andrei*	Bacteroidetes	Sphingobacteriia	Sphingobacteriales	NS11-12_marine_group	unclassified	0.84	0.06
ASV40	bedding	*Eisenia andrei*	Bacteroidetes	Cytophagia	Cytophagales	Cytophagaceae	unclassified	0.83	0.07
ASV45	bedding	*Eisenia andrei*	Bacteroidetes	Cytophagia	Cytophagales	Cytophagaceae	*Leadbetterella*	0.81	0.07
**ASV1**	**cocoon**	**Eisenia fetida**	**Proteobacteria**	**Betaproteobacteria**	**Burkholderiales**	**Comamonadaceae**	***Verminephrobacter***	**44.68**	**4.77**
**ASV3**	**cocoon**	**Eisenia fetida**	**Actinobacteria**	**Actinobacteria**	**Micrococcales**	**Microbacteriaceae**	**unclassified**	**16.57**	**2.72**
ASV6	cocoon	Eisenia fetida	Proteobacteria	Alphaproteobacteria	Rhizobiales	Hyphomicrobiaceae	*Devosia*	6.72	3.59
ASV5	cocoon	Eisenia fetida	Proteobacteria	Alphaproteobacteria	Rhodospirillales	unclassified	unclassified	5.38	5.37
ASV7	cocoon	Eisenia fetida	Proteobacteria	Alphaproteobacteria	Rhizobiales	Rhizobiaceae	*Neorhizobium*	3.79	3.78
ASV8	cocoon	Eisenia fetida	Proteobacteria	Alphaproteobacteria	Rhodospirillales	Rhodospirillaceae	*Ferrovibrio*	3.59	1.54
**ASV11**	**cocoon**	**Eisenia fetida**	**Bacteroidetes**	**Cytophagia**	**Cytophagales**	**Cytophagaceae**	***Candidatus_Nephrothrix***	**2.13**	**0.88**
ASV17	cocoon	Eisenia fetida	Proteobacteria	Gammaproteobacteria	Xanthomonadales	Xanthomonadaceae	unclassified	1.92	1.05
**ASV4**	**cocoon**	**Eisenia fetida**	**Bacteroidetes**	**Cytophagia**	**Cytophagales**	**Cytophagaceae**	***Candidatus_Nephrothrix***	**1.70**	**1.38**
**ASV12**	**cocoon**	**Eisenia fetida**	**Bacteroidetes**	**Cytophagia**	**Cytophagales**	**Cytophagaceae**	***Candidatus_Nephrothrix***	**1.65**	**0.75**
ASV15	cocoon	Eisenia fetida	Proteobacteria	Alphaproteobacteria	Rhizobiales	Rhodobiaceae	*Parvibaculum*	1.37	1.22
ASV18	cocoon	Eisenia fetida	Proteobacteria	Alphaproteobacteria	Rhizobiales	Hyphomicrobiaceae	*Devosia*	1.36	1.18
ASV37	cocoon	Eisenia fetida	Bacteroidetes	Sphingobacteriia	Sphingobacteriales	Chitinophagaceae	*Taibaiella*	0.66	0.56
ASV46	cocoon	Eisenia fetida	Proteobacteria	Alphaproteobacteria	Rhizobiales	Rhizobiaceae	*Rhizobium*	0.50	0.26
**ASV41**	**cocoon**	**Eisenia fetida**	**Bacteroidetes**	**Cytophagia**	**Cytophagales**	**Cytophagaceae**	***Candidatus_Nephrothrix***	**0.47**	**0.44**
ASV65	cocoon	Eisenia fetida	Proteobacteria	Alphaproteobacteria	Rhodospirillales	Rhodospirillaceae	*Ferrovibrio*	0.42	0.21
ASV63	cocoon	Eisenia fetida	Proteobacteria	Gammaproteobacteria	Pseudomonadales	Pseudomonadaceae	*Pseudomonas*	0.29	0.18
ASV72	cocoon	Eisenia fetida	Proteobacteria	Alphaproteobacteria	Rhizobiales	Rhodobiaceae	*Parvibaculum*	0.28	0.28
ASV110	cocoon	Eisenia fetida	Proteobacteria	Deltaproteobacteria	Myxococcales	Nannocystaceae	unclassified	0.25	0.24
ASV52	cocoon	Eisenia fetida	Proteobacteria	Alphaproteobacteria	Rhizobiales	Hyphomicrobiaceae	*Devosia*	0.22	0.18
ASV9	bedding	Eisenia fetida	Proteobacteria	Gammaproteobacteria	Pseudomonadales	Moraxellaceae	*Acinetobacter*	3.62	1.64
ASV23	bedding	Eisenia fetida	Proteobacteria	Gammaproteobacteria	Enterobacteriales	Enterobacteriaceae	unclassified	2.20	1.04
ASV21	bedding	Eisenia fetida	Proteobacteria	Alphaproteobacteria	Rhodospirillales	Acetobacteraceae	*Acetobacter*	2.18	0.96
ASV20	bedding	Eisenia fetida	Proteobacteria	Gammaproteobacteria	Enterobacteriales	Enterobacteriaceae	unclassified	2.12	0.81
ASV24	bedding	Eisenia fetida	Proteobacteria	Alphaproteobacteria	Rhodospirillales	Acetobacteraceae	*Acetobacter*	1.63	0.76
ASV34	bedding	Eisenia fetida	Bacteroidetes	Flavobacteriia	Flavobacteriales	Flavobacteriaceae	*Flavobacterium*	1.20	0.56
ASV38	bedding	Eisenia fetida	Bacteroidetes	Cytophagia	Cytophagales	Cytophagaceae	*Chryseolinea*	1.00	0.23
ASV43	bedding	Eisenia fetida	Bacteroidetes	Flavobacteriia	Flavobacteriales	Flavobacteriaceae	*Flavobacterium*	0.91	0.36
ASV42	bedding	Eisenia fetida	Proteobacteria	Gammaproteobacteria	Pseudomonadales	Moraxellaceae	*Acinetobacter*	0.83	0.39
ASV53	bedding	Eisenia fetida	Proteobacteria	Gammaproteobacteria	Enterobacteriales	Enterobacteriaceae	unclassified	0.66	0.32
ASV54	bedding	Eisenia fetida	Bacteroidetes	Sphingobacteriia	Sphingobacteriales	unclassified	unclassified	0.63	0.24
ASV62	bedding	Eisenia fetida	Proteobacteria	Gammaproteobacteria	Pseudomonadales	Pseudomonadaceae	*Pseudomonas*	0.60	0.14
ASV97	bedding	Eisenia fetida	Proteobacteria	Epsilonproteobacteria	Campylobacterales	Campylobacteraceae	*Arcobacter*	0.55	0.34
ASV69	bedding	Eisenia fetida	Proteobacteria	Deltaproteobacteria	Myxococcales	Nannocystaceae	*Nannocystis*	0.54	0.26
ASV68	bedding	Eisenia fetida	Proteobacteria	Gammaproteobacteria	Pseudomonadales	Pseudomonadaceae	*Pseudomonas*	0.52	0.19
ASV96	bedding	Eisenia fetida	Bacteroidetes	Cytophagia	Cytophagales	Cytophagaceae	*Chryseolinea*	0.44	0.17
ASV98	bedding	Eisenia fetida	Bacteroidetes	Sphingobacteriia	Sphingobacteriales	env.OPS_17	unclassified	0.41	0.19
ASV100	bedding	Eisenia fetida	Chlamydiae	Chlamydiae	Chlamydiales	cvE6	unclassified	0.41	0.12
ASV89	bedding	Eisenia fetida	Proteobacteria	Gammaproteobacteria	Xanthomonadales	Xanthomonadaceae	unclassified	0.40	0.28
ASV83	bedding	Eisenia fetida	Proteobacteria	Alphaproteobacteria	Rhizobiales	Rhizobiaceae	unclassified	0.39	0.10

Supplementary Table 2. Mean relative abundance (%) of amplicon sequence variants (ASVs) found in bacterial communities of cocoons and bedding from the earthworm species Eisenia andrei and E. fetida whose taxonomy matchs those of described bacteria found in earthworm nephridia (ref 14,15). ASVs marked in bold are possible nephridial symbionts

	Eisenia andrei		*Eisenia fetida*	
Amplicon sequence variant (ASV)	cocoon	bedding	cocoon	bedding
**ASV25, *Achromobacter***	**0.893**	**0.000**	**0.015**	**0.168**
**ASV228, *Achromobacter***	**0.002**	**0.149**	**0.000**	**0.047**
**ASV1246, *Azospira***	**0.015**	**0.015**	**0.000**	**0.000**
**ASV370,*Azospirillum***	**0.001**	**0.133**	**0.000**	**0.000**
ASV2172, *Bordetella*	0	0	0	0.009
**ASV420, *Bordetella***	**0.001**	**0.094**	**0.000**	**0.024**
ASV3622, *Bosea*	0	0	0	0.006
**ASV1186, *Bosea***	**0.000**	**0.000**	**0.016**	**0.000**
ASV2417, *Bosea*	0	0.005	0	0.000
ASV668, *Bosea*	0	0	0	0.061
**ASV506, *Bosea***	**0.041**	**0.015**	**0.000**	**0.000**
**ASV310, *Bradyrhizobium***	**0.000**	**0.015**	**0.004**	**0.120**
**ASV10, *Chitinophaga***	**0.041**	**4.532**	**0.006**	**0.124**
ASV2376, *Chitinophaga*	0	0	0	0.012
ASV2630,, *Chitinophaga*	0	0	0	0.007
ASV5306, *Chitinophaga*	0	0	0	0.004
ASV246, *Chitinophaga*	0	0	0	0.185
ASV938, *Chitinophaga*	0	0.044	0	0
ASV1078, , *Chitinophaga*	0	0.039	0	0.004
ASV3000, *Chitinophaga*	0	0	0	0.008
**ASV388, , *Chitinophaga***	**0.069**	**0.003**	**0**	**0.000**
**ASV708, *Chitinophaga***	**0.014**	**0.045**	**0**	**0.003**
ASV378, *Chitinophaga*	0	0	0	0.076
**ASV1109, *Chitinophaga***	**0.013**	**0.010**	**0**	**0.000**
ASV914, *Chitinophaga*	0	0	0	0.039
ASV6129, *Chitinophaga*	0	0.002	0	0
ASV4127, *Chitinophaga*	0	0	0	0.005
ASV173, *Chitinophaga*	0	0.208	0	0.023
ASV5006, *Chitinophaga*	0	0	0	0.002
ASV596, *Desulfovibrio*	0	0.056	0	0.003
ASV2627, *Devosia*	0	0	0	0.009
**ASV4477, *Devosia***	**0.002**	**0.000**	**0**	**0.000**
**ASV850, *Devosia***	**0.001**	**0.000**	**0.001**	**0.043**
ASV2083, *Devosia*	0	0	0	0.016
ASV2606, *Devosia*	0	0	0.005	0
ASV2836, *Devosia*	0	0	0.004	0
**ASV52, *Devosia***	**0.105**	**0.177**	**0.222**	**0.023**
**ASV1157, *Devosia***	**0**	**0**	**0.017**	**0**
**ASV18, *Devosia***	**0.083**	**0.105**	**1.361**	**0.098**
**ASV299, *Devosia***	**0.096**	**0**	**0**	**0**
**ASV1546, *Devosia***	**0.000**	**0**	**0.013**	**0**
**ASV6, *Devosia***	**0.055**	**0**	**6.721**	**0.028**
**ASV1104, *Devosia***	**0.000**	**0**	**0.019**	**0.000**
ASV2077, *Devosia*	0	0	0	0.008
ASV5217, *Devosia*	0	0	0.002	0
**ASV1317, *Devosia***	**0**	**0**	**0.002**	**0.022**
**ASV66, *Devosia***	**0**	**0.126**	**0.095**	**0.308**
**ASV356, *Devosia***	**0.069**	**0.003**	**0**	**0**
**ASV1405, *Devosia***	**0**	**0**	**0.011**	**0**
**ASV133, *Devosia***	**0.162**	**0.089**	**0.008**	**0.020**
**ASV389, *Devosia***	**0.034**	**0.049**	**0**	**0**
**ASV2326, *Devosia***	**0**	**0.003**	**0.001**	**0**
**ASV566, *Devosia***	**0**	**0**	**0.037**	**0.005**
**ASV1706, *Devosia***	**0**	**0**	**0.006**	**0.001**
**ASV1796, *Devosia***	**0**	**0**	**0.008**	**0**
**ASV179, *Devosia***	**0**	**0**	**0.134**	**0**
**ASV1603, *Devosia***	**0**	**0**	**0.012**	**0.005**
ASV324, *Devosia*	0	0	0	0.120
**ASV3003, *Devosia***	**0.003**	**0**	**0**	**0**
ASV3149, *Dyadobacter*	0	0.005	0	0.003
**ASV1831, *Dyadobacter***	**0**	**0**	**0.012**	**0.000**
**ASV1091, *Dyadobacter***	**0**	**0**	**0.008**	**0.018**
ASV4852, *Dyadobacter*	0	0	0	0.002
ASV3488, *Dyadobacter*	0	0	0	0.013
ASV5338, *Dyadobacter*	0	0	0	0.002
ASV2598, *Dyadobacter*	0	0	0	0.006
**ASV1653, *Dyadobacter***	**0.006**	**0**	**0**	**0.015**
**ASV633, *Dyadobacter***	**0**	**0**	**0.003**	**0.065**
**ASV460, *Dyadobacter***	**0**	**0**	**0.006**	**0.096**
**ASV1229, *Dyadobacter***	**0**	**0**	**0.010**	**0**
ASV481, *Dyadobacter*	0	0.069	0	0.025
**ASV2922, *Dyadobacter***	**0.002**	**0.000**	**0**	**0.002**
ASV964, *Dyadobacter*	0	0	0	0.047
ASV1746, *Dyadobacter*	0	0.012	0	0
ASV5014, *Dyadobacter*	0	0	0	0.003
ASV4534, *Flavisolibacter*	0	0	0	250.000
ASV2432, *Flexibacter*	0	0	0	0.009
ASV2482, *Flexibacter*	0	0	0	0.008
ASV292, *Klebsiella*	0	0	0	0.144
ASV2206, *Mesorhizobium*	0	0	0	0.006
ASV3756, *Mesorhizobium*	0	0	0.004	0
**ASV1583, *Mesorhizobium***	**0.003**	**0.011**	**0**	**0**
**ASV259, *Mesorhizobium***	**0**	**0**	**0.017**	**0.100**
**ASV3678, *Mesorhizobium***	**0.003**	**0**	**0**	**0**
**ASV1533, *Mesorhizobium***	**0**	**0**	**0.010**	**0**
**ASV2272, *Methylophilus***	**0**	**0**	**0.001**	**0.009**
ASV2745, *Ochrobactrum*	0	0	0	0.005
ASV348, *Ochrobactrum*	0	0.096	0	0.035
ASV2123, *Ochrobactrum*	0	0.010	0	0.006
**ASV532, *Paenibacillus***	**0.010**	**0**	**0.029**	**0.000**
ASV2851, *Paenibacillus*	0	0	0	0.007
ASV6193, *Paenibacillus*	0	0	0	0.002
ASV4571, *Paenibacillus*	0	0	0	0.002
ASV3109, *Paenibacillus*	0	0	0	0.011
**ASV1355, *Paenibacillus***	**0**	**0**	**0.008**	**0.013**
ASV6105, *Paenibacillus*	0	0.002	0	0
ASV1356, *Paenibacillus*	0	0.039	0	0.002
**ASV2879, *Paenibacillus***	**0**	**0**	**0.002**	**0.004**
ASV5513, *Paenibacillus*	0	0	0	0.003
**ASV3496, *Paenibacillus***	**0.003**	**0.004**	**0**	**0**
ASV2254, *Paenibacillus*	0	0	0	0.013

	Eisenia andrei		*Eisenia fetida*	
Amplicon sequence variant (ASV)	cocoon	bedding	cocoon	bedding
ASV4276, *Paenibacillus*	0	0	0	0.002
**ASV942, *Paenibacillus***	**0.003**	**0.043**	**0**	**0.001**
ASV3940, *Paenibacillus*	0	0.003	0	0
ASV3613, *Paenibacillus*	0	0.000	0	0.005
**ASV3048, *Paenibacillus***	**0**	**0.000**	**0.004**	**0.000**
ASV6128, *Paenibacillus*	0	0.002	0	0
ASV875, *Paenibacillus*	0	0.043	0	0.010
ASV3773, *Paenibacillus*	0	0.010	0	0
**ASV1227, *Paenibacillus***	**0.009**	**0.013**	**0**	**0.000**
ASV1057, *Paenibacillus*	0	0	0	0.033
ASV2201, *Paenibacillus*	0	0	0	0.010
ASV1387, *Paenibacillus*	0	0	0	0.023
ASV511, *Paenibacillus*	0	0	0	0.064
ASV3642, *Paenibacillus*	0	0	0	0.003
ASV2238, *Paenibacillus*	0	0	0	0.008
ASV3669, *Paenibacillus*	0	0	0	0.005
ASV2389, *Paenibacillus*	0	0.010	0	0
ASV3098, *Paenibacillus*	0	0.007	0	0
**ASV672, *Paenibacillus***	**0**	**0**	**0.033**	**0**
**ASV1889, *Paenibacillus***	**0**	**0**	**0.009**	**0**
ASV3463, *Paenibacillus*	0	0	0	0.004
ASV2331, *Paenibacillus*	0	0.009	0	0
ASV1514, *Paenibacillus*	0	0.036	0	0
ASV2975, *Paenibacillus*	0	0.010	0	0
ASV3027, *Paenibacillus*	0	0	0	0.008
**ASV1174, *Paenibacillus***	**0.001**	**0.024**	**0**	**0.004**
**ASV1999, *Paenibacillus***	**0.005**	**0.009**	**0**	**0**
**ASV1089, *Paenibacillus***	**0.001**	**0.009**	**0**	**0.024**
ASV2670, *Paenibacillus*	0	0	0	0.005
ASV3459, *Paenibacillus*	0	0	0	0.005
ASV3866, *Paenibacillus*	0	0	0	0.003
**ASV5428, *Paenibacillus***	**0**	**0**	**0.002**	**0**
ASV1044, *Pedobacter*	0	0.039	0	0
**ASV788, *Pedobacter***	**0**	**0**	**0.005**	**0.042**
**ASV242, *Pedobacter***	**0.065**	**0.054**	**0**	**0.029**
**ASV581, *Pedobacter***	**0**	**0**	**0.053**	**0.006**
**ASV1874, *Pedobacter***	**0.008**	**0**	**0**	**0**
**ASV680, *Pedobacter***	**0.049**	**0**	**0**	**0**
ASV311, *Pedobacter*	0	0	0	0.137
**ASV84, *Pedobacter***	**0.031**	**0.523**	**0.000**	**0.005**
**ASV301, *Pedobacter***	**0**	**0**	**0.084**	**0**
**ASV387, *Pedobacter***	**0**	**0.120**	**0.002**	**0**
**ASV552, *Pedobacter***	**0**	**0**	**0.041**	**0.028**
ASV4589, *Pedobacter*	0	0	0	0.004
**ASV1688, *Pedobacter***	**0**	**0**	**0.009**	**0**
ASV434, *Pedobacter*	0	0.101	0	0.008
ASV4566, *Pedobacter*	0	0	0	0.002
ASV3822, *Pedobacter*	0	0	0	0.009
ASV3725, *Pedobacter*	0	0	0	0.004
ASV3460, *Pedobacter*	0	0	0	0.005
**ASV4902, *Pedobacter***	**0**	**0**	**0.002**	**0**
**ASV5413, *Phyllobacterium***	**0**	**0**	**0.002**	**0**
**ASV29, *Pseudomonas***	**0.042**	**0.894**	**0.098**	**0.348**
ASV236, *Pseudomonas*	0	0.221	0	0
**ASV1467, *Pseudomonas***	**0**	**0**	**0.013**	**0**
ASV1607, *Pseudomonas*	0	0.016	0	0.000
**ASV62, *Pseudomonas***	**0**	**0**	**0.010**	**0.595**
ASV840, *Pseudomonas*	0	0	0	0.047
ASV2912, *Pseudomonas*	0	0	0	0.008
ASV1588, *Pseudomonas*	0	0.022	0	0.009
ASV3190, *Pseudomonas*	0	0.005	0	0
ASV156, *Pseudomonas*	0	0.255	0	0.075
ASV932, *Pseudomonas*	0	0.040	0	0
**ASV2118, *Pseudomonas***	**0**	**0**	**0.006**	**0.006**
ASV275, *Pseudomonas*	0	0.027	0	0.129
ASV519, *Pseudomonas*	0	0.098	0	0
ASV901, *Pseudomonas*	0	0	0	0.041
**ASV82, *Pseudomonas***	**0**	**0.050**	**0.075**	**0.337**
**ASV293, *Pseudomonas***	**0**	**0**	**0.024**	**0.089**
**ASV81, *Pseudomonas***	**0**	**0.045**	**0.080**	**0.331**
**ASV63, *Pseudomonas***	**0**	**0**	**0.292**	**0.115**
**ASV68, *Pseudomonas***	**0**	**0**	**0.015**	**0.521**
**ASV822, *Pseudomonas***	**0**	**0.042**	**0.012**	**0**
**ASV393, *Pseudomonas***	**0**	**0**	**0.003**	**0.088**
ASV2993, *Pseudomonas*	0	0	0	0.002
**ASV224, *Pseudomonas***	**0**	**0**	**0.091**	**0**
**ASV104, *Rhizobium***	**0**	**0.040**	**0.087**	**0.211**
ASV283, *Rhizobium*	0	0.053	0.000	0.118
**ASV2325, *Rhizobium***	**0**	**0**	**0.005**	**0**
**ASV4757, *Rhizobium***	**0**	**0**	**0.002**	**0**
**ASV374, *Rhizobium***	**0.001**	**0.006**	**0.012**	**0.075**
**ASV46, *Rhizobium***	**0**	**0**	**0.501**	**0.047**
**ASV2382, *Rhizobium***	**0**	**0**	**0.006**	**0**
**ASV1464, *Sphingomonas***	**0.002**	**0**	**0.004**	**0**
**ASV4744, *Sphingomonas***	**0.002**	**0**	**0**	**0**
ASV1596, *Sphingomonas*	0	0.013	0	0.012
ASV1188, *Sphingomonas*	0	0.019	0	0.002
ASV6149, *Sphingomonas*	0	0.002	0	0
**ASV1256, *Sphingomonas***	**0**	**0**	**0.019**	**0.000**
ASV1486, *Sphingomonas*	0	0	0	0.021
ASV1860, *Sphingomonas*	0	0	0	0.013
ASV3446, *Sphingomonas*	0	0	0	0.002
**ASV1087, *Sphingomonas***	**0.004**	**0.028**	**0**	**0**
ASV4257, *Sphingomonas*	0	0	0	0.004
ASV480, *Stenotrophomonas*	0	0.111	0	0
ASV1984, *Stenotrophomonas*	0	0	0	0.020
**ASV1026, *Stenotrophomonas***	**0**	**0.018**	**0.002**	**0.013**
ASV287, *Stenotrophomonas*	0	0.167	0	0.008
ASV595, *Stenotrophomonas*	0	0.069	0	0
**ASV168, *Stenotrophomonas***	**0.025**	**0.260**	**0**	**0**
ASV5199, *Stenotrophomonas*	0	0	0	0.002

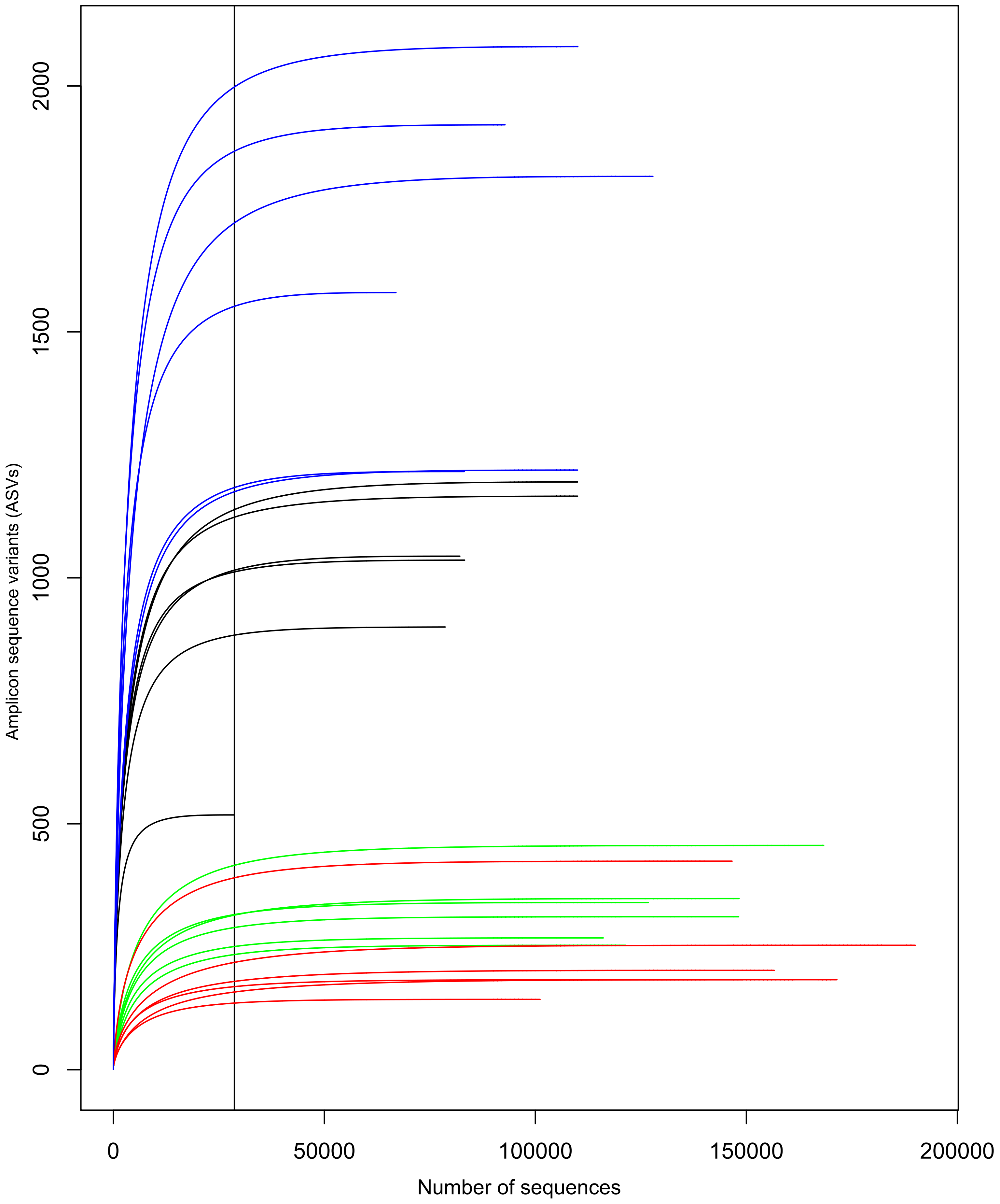

Supplementary Figure 1. Rarefaction curves indicating the number of amplicon sequence variants (ASVs) identified, at a genetic distance of 3%, in bacterial communities in cocoons of *Eisenia andrei* (green) and *Eisenia fetida* (red) and in the respective vermicompost beds (*E. andrei*, black and *E. fetida*, blue).

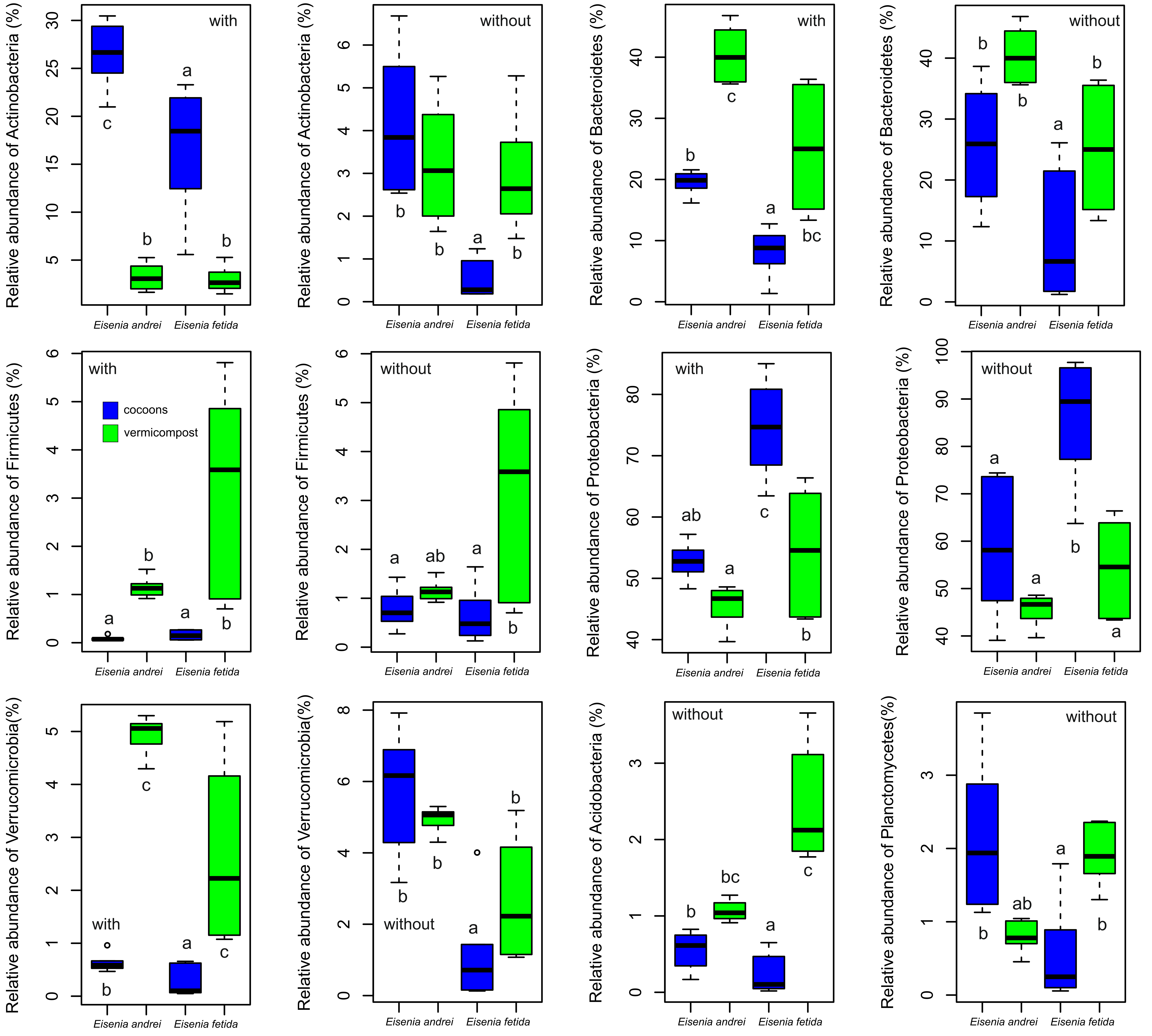

Suplementary Figure 2. Box plots of the relative abundance of bacterial phyla in cocoons (blue) of the earthworm species *Eisenia andrei* and *E. fetida* and in the respective vermicompost (green) samples. Different letters indicate significant differences between treatments (Tukey HSD test, FDR corrected). Figures labelled "with" show relative abundances including the three nephridial bacterial symbionts *(Verminephrobacter, Candidatus Nephrothrix* and Microbacteriaceae), those figures labelled "without" show relative abundances without the three symbionts.

ANOVA table analyzing the effect of earthworm species *(Eisenia andrei* and *Eisenia fetida*) and type of sample (cocoon and bedding)and its interaction on relative abundance of most abundant bacterial phyla with or without the known vertical transmitted bacterial symbionts *(Verminephrobacter, Candidatus Nephrothrix* and Microbacteriaceae). We give F and P values (P<0.0001 ***, P<0.001 ** and P<0.05 *).

		Actinobacteria	*Bacteroidetes*	*Firmicutes*	*Proteobacteria*	*Verrucomicrobia*	*Acidobacteria*	*Planctomycetes*
without symbionts	$Species_{1,20}$	13.82**	16.15***	4.70*	15.49***	32.25***	8.99**	0.81
	$Type_{1,20}$	2.07	14.81**	10.69**	23.23***	0.29	60.13***	0.004
	Species x $Type_{1,20}$	10.33**	0.001	5.97*	4.01	3.66	23.26***	25.18***
with symbionts	$Species_{1,20}$	9.84**	29.74***	6.30*	27.67***	12.11**		
	$Type_{1,20}$	133.84***	59.68***	23.26***	22.10***	78.75**		
	Species x $Type_{1,20}$	8.84**	0.67	5.50*	4.92**	6.32*		

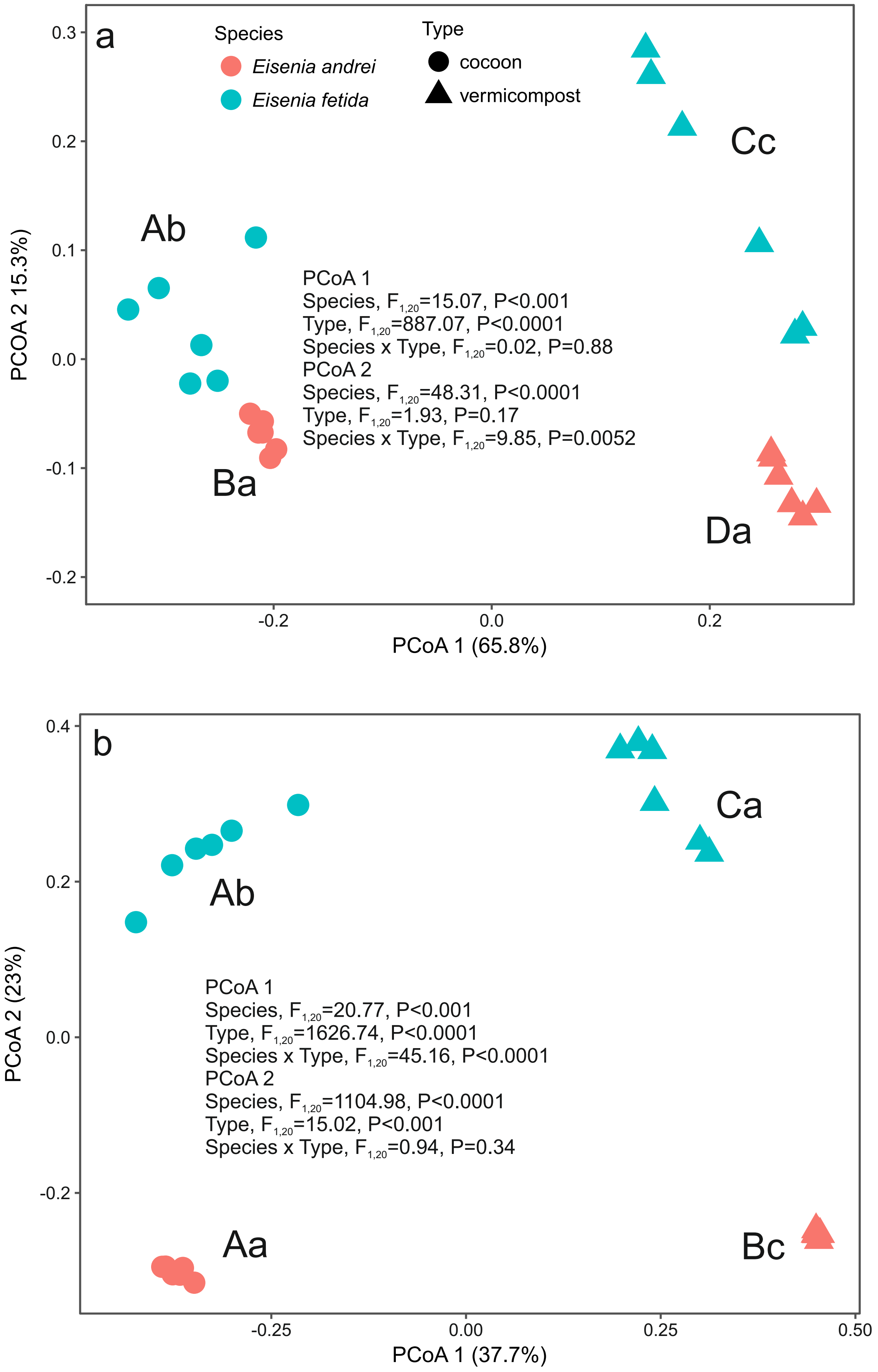

Supplementary Figure 3. . Principal coordinate analyses (PCoAs) of (a)weighted UniFrac and (b) Bray-Curtis β-diversity of bacterial communities in cocoons of the earthworm species *Eisenia andrei* and *E. fetida* and in the respective vermicompost beds. Different capital and lower case letters indicate significant differences between treatments in PCoA 1 and 2 scores respectively (Tukey HSD test, FDR

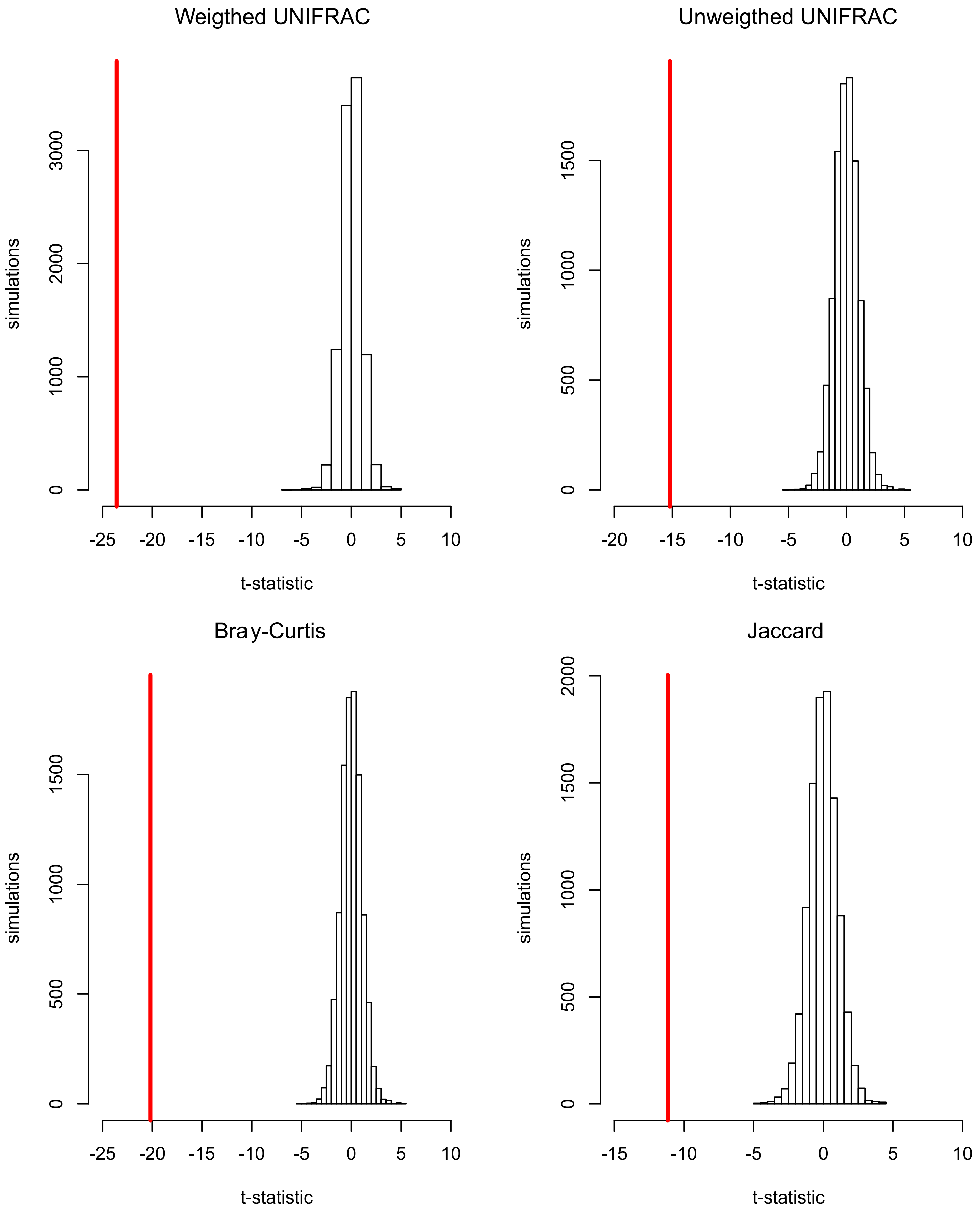

Supplementary Figure 4. Distribution of Monte Carlo simulations (n = 10000) of the t-statistic values for the original data (see red lines) and the t-statistic values for randomly allocated observations; weighted Unifrac: t = -23.58, *P*=0; unweighted Unifrac : t = -15.21, *P*=0; Bray-Curtis : t=-20.17, *P*=0; and Jaccard : t = -11.16, *P*=0. *P*-values were calculated as the probability of randomized t-test values exceeding the distribution of t-test values based on original data.

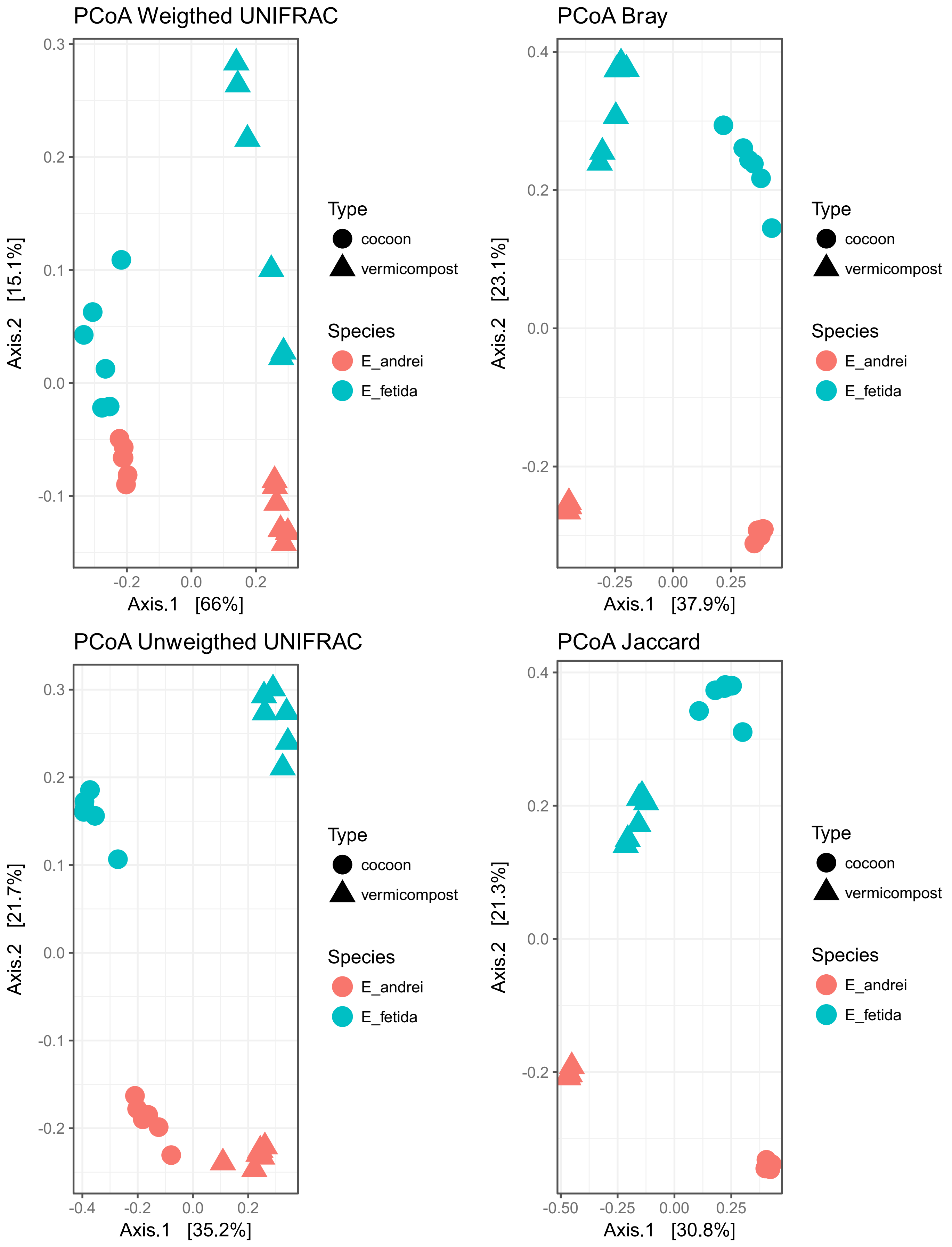

Supplementary Figure 5. . Principal coordinate analyses (PCoAs) of weighted and unweighted UniFrac, Jaccard and Bray-Curtis β-diversity of bacterial communities in cocoons of the earthworm species *Eisenia andrei* and *E. fetida* and in the respective vermicompost beds. For this analysis we used only the most abundant ASVs (relative abundance > 0.0001) shifting from a matrix with 5411

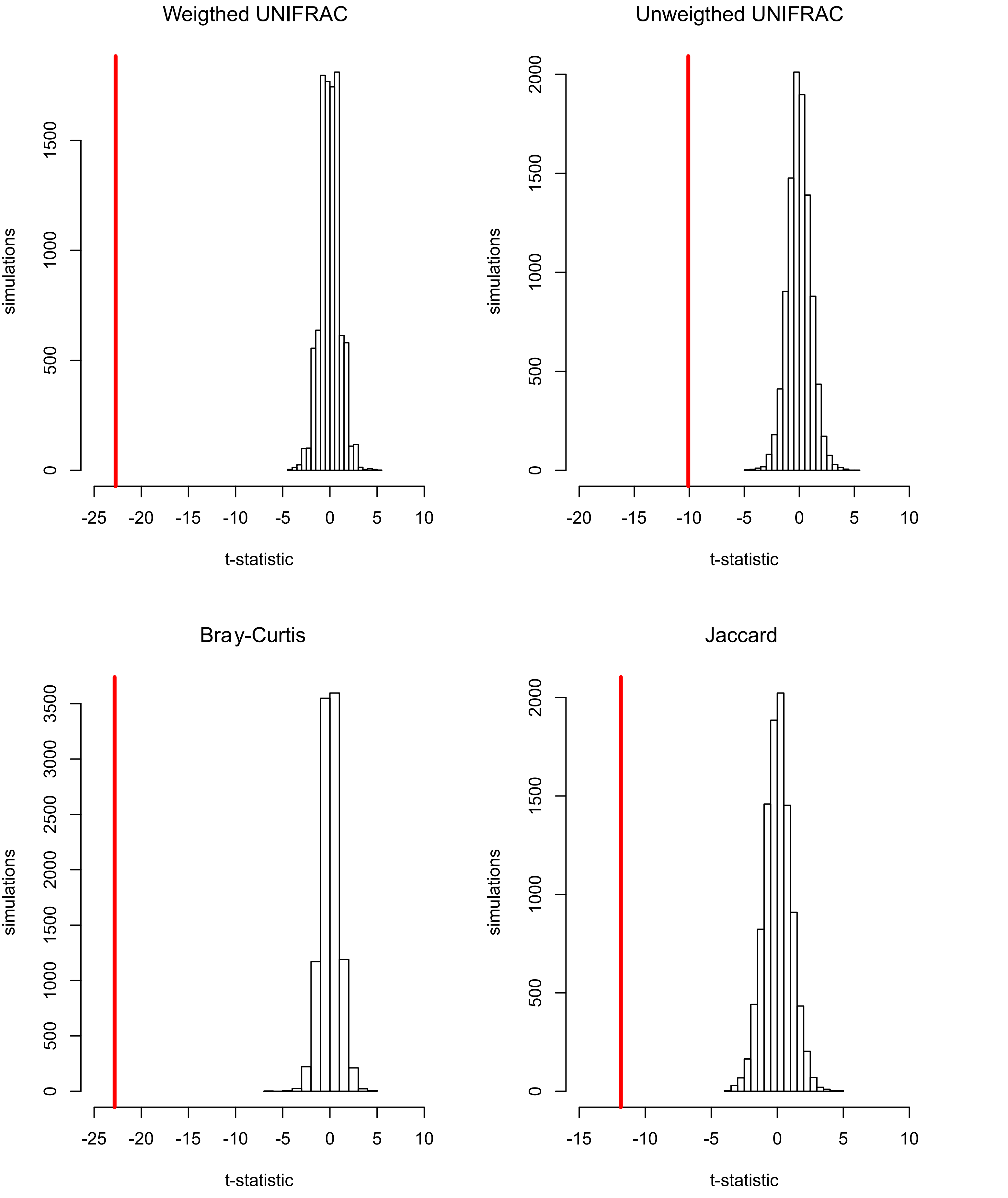

Supplementary Figure 6. Distribution of Monte Carlo simulations (n = 10000) of the t-statistic values for the original data (see red lines) and the t-statistic values for randomly allocated observations for the most abundant OTUs (see supplementary figure 5). Weighted Unifrac: t = -22.72, *P*=0; unweighted Unifrac: t = -10.08, *P*=0; Bray-Curtis : t=-22.83, *P*=0; and Jaccard : t = -11.85, *P*=0. *P*-values were calculated as the probability of randomized t-test values exceeding the distribution of t-test values based on original data.

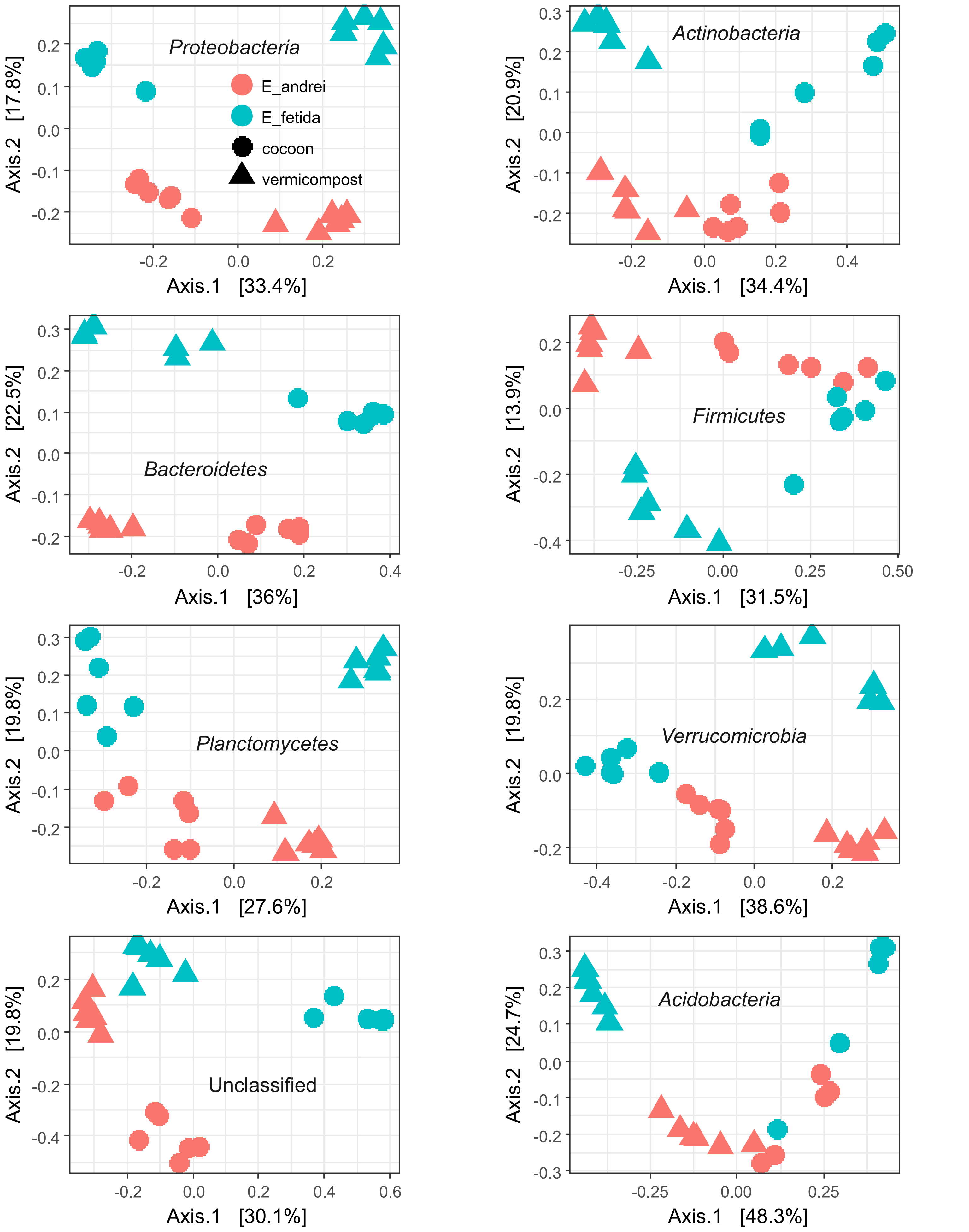

Supplementary Figure 7. . Principal coordinate analyses (PCoAs) of unweighted UNIFRAC distances for each of the most abundant bacterial phyla in the bacterial communities in cocoons of the earthworm species *Eisenia andrei* and *E. fetida* and in the respective bedding materials.

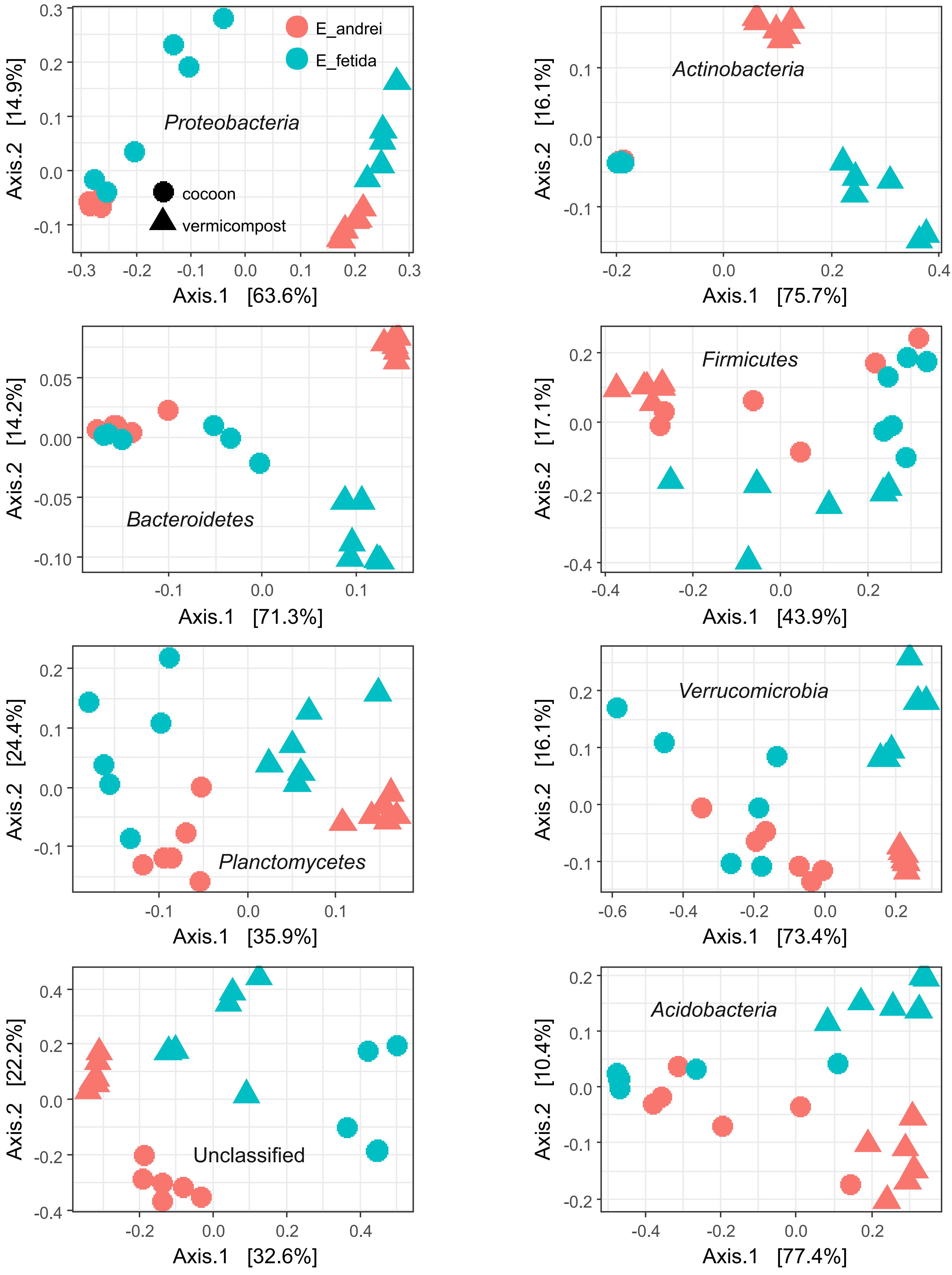

Supplementary Figure 8. . Principal coordinate analyses (PCoAs) of weighted UNIFRAC distances for each of the most abundant bacterial phyla in the bacterial communities in cocoons of the earthworm species *Eisenia andrei* and *E. fetida* and in the respective bedding materials.

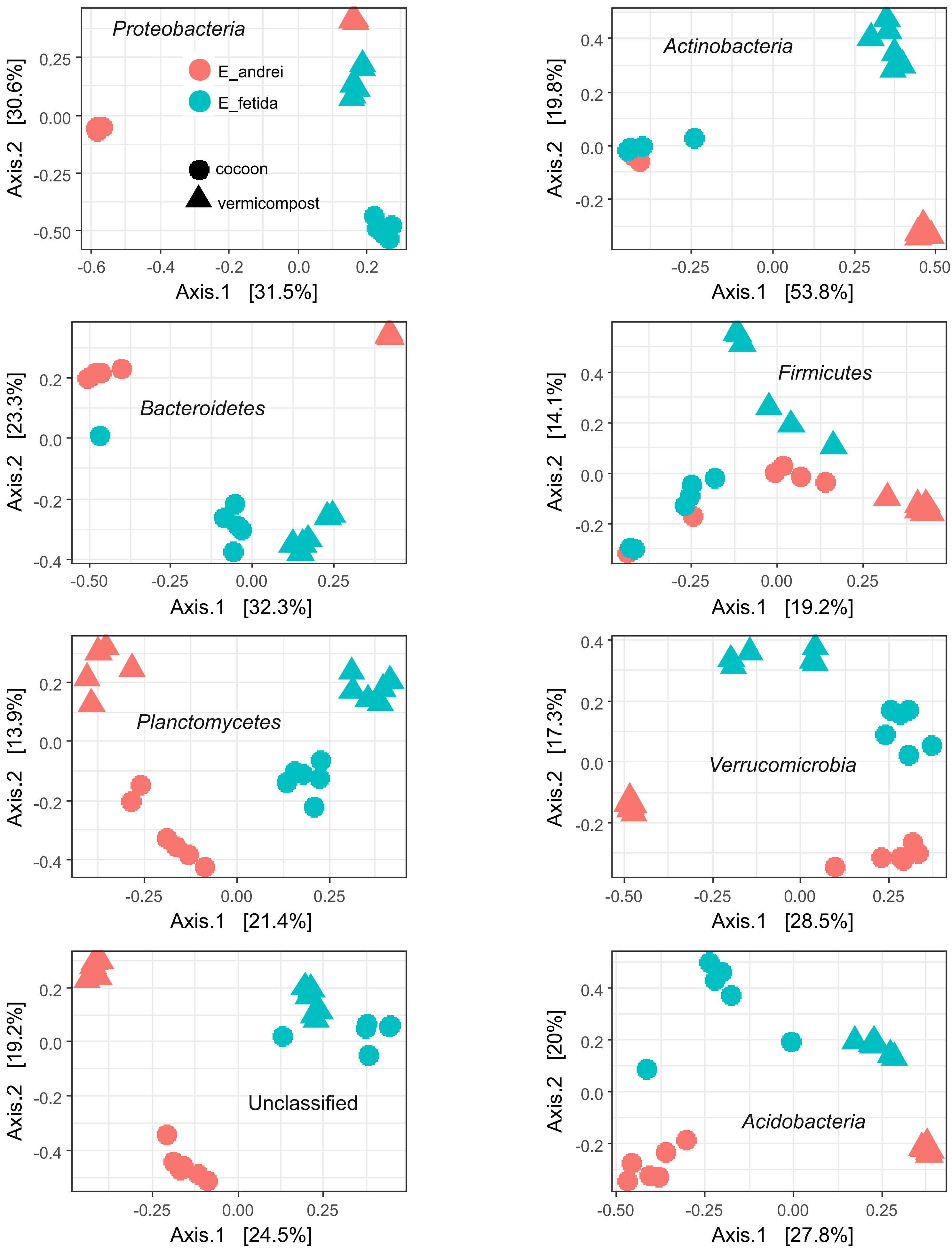

Supplementary Figure 9. . Principal coordinate analyses (PCoAs) of Bray-Curtis distances for each of the most abundant bacterial phyla in the bacterial communities in cocoons of the earthworm species *Eisenia andrei* and *E. fetida* and in the respective bedding materials.

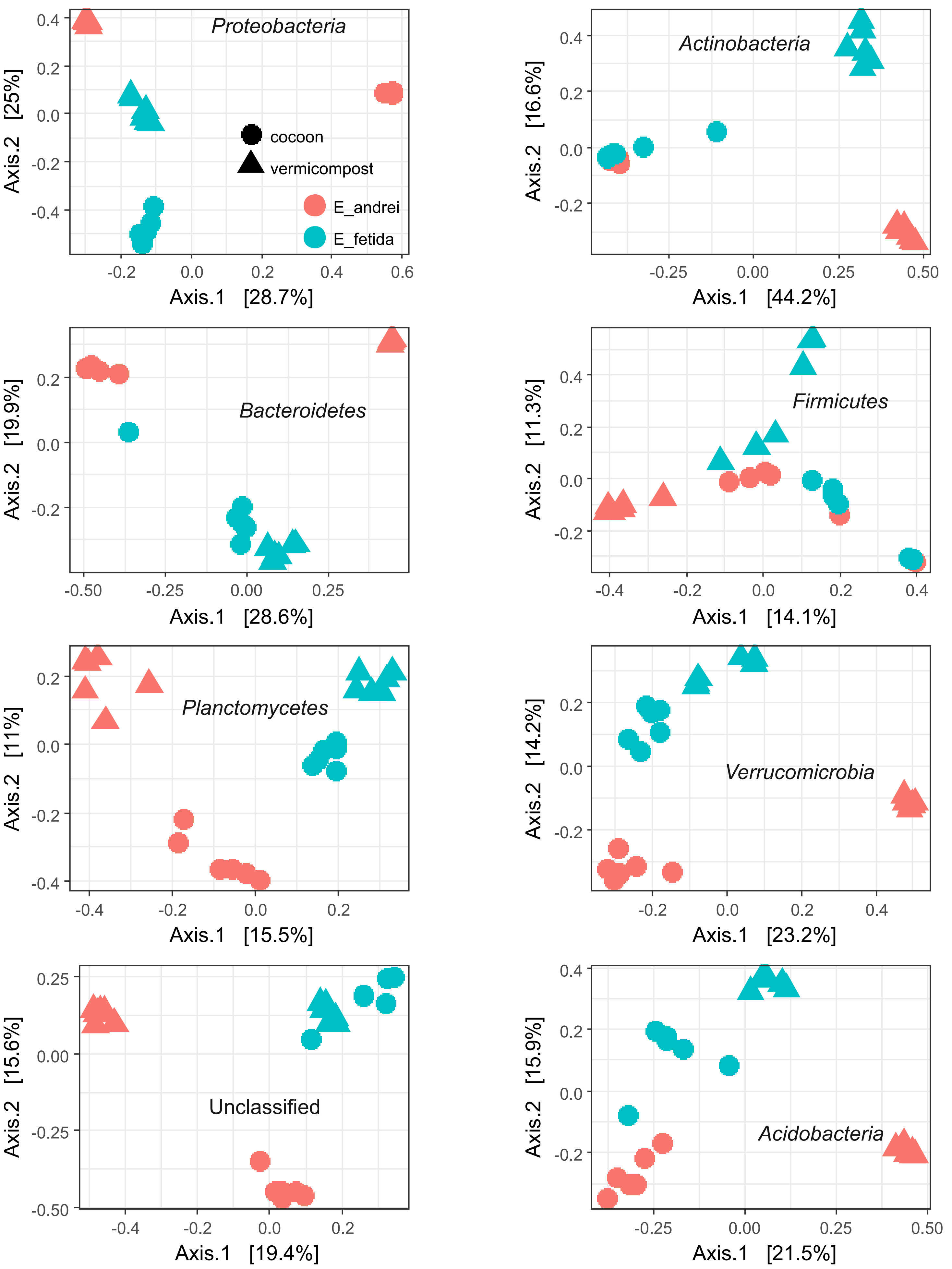

Supplementary Figure 10. . Principal coordinate analyses (PCoAs) of Jaccard distances for each of the most abundant bacterial phyla in the bacterial communities in cocoons of the earthworm species *Eisenia andrei* and *E. fetida* and in the respective bedding materials.
